# Analysis of companion cell and phloem metabolism using a transcriptome-guided model of Arabidopsis metabolism

**DOI:** 10.1093/plphys/kiad154

**Published:** 2023-03-11

**Authors:** Hilary Hunt, Nico Brueggen, Alexander Galle, Sandy Vanderauwera, Claus Frohberg, Alisdair R Fernie, Uwe Sonnewald, Lee J Sweetlove

**Affiliations:** Department of Plant Sciences, University of Oxford, South Parks Rd, Oxford OX1 3RB, UK; Department of Biology, Division of Biochemistry, Friedrich–Alexander University Erlangen–Nürnberg, Staudtstrasse 5, Erlangen 91ß58, Germany; BASF Belgium Coordination Center CommV, Innovation Center Gent, Technologiepark-Zwijnaarde 101, Gent 9052, Belgium; BASF Belgium Coordination Center CommV, Innovation Center Gent, Technologiepark-Zwijnaarde 101, Gent 9052, Belgium; BASF Belgium Coordination Center CommV, Innovation Center Gent, Technologiepark-Zwijnaarde 101, Gent 9052, Belgium; Max Planck Institute of Molecular Plant Physiology, Am Mühlenberg 1, Potsdam 14476, Germany; Department of Biology, Division of Biochemistry, Friedrich–Alexander University Erlangen–Nürnberg, Staudtstrasse 5, Erlangen 91ß58, Germany; Department of Plant Sciences, University of Oxford, South Parks Rd, Oxford OX1 3RB, UK

## Abstract

Companion cells and sieve elements play an essential role in vascular plants, and yet the details of the metabolism that underpins their function remain largely unknown. Here, we construct a tissue-scale flux balance analysis (FBA) model to describe the metabolism of phloem loading in a mature Arabidopsis (*Arabidopsis thaliana*) leaf. We explore the potential metabolic interactions between mesophyll cells, companion cells, and sieve elements based on the current understanding of the physiology of phloem tissue and through the use of cell type–specific transcriptome data as a weighting in our model. We find that companion cell chloroplasts likely play a very different role to mesophyll chloroplasts. Our model suggests that, rather than carbon capture, the most crucial function of companion cell chloroplasts is to provide photosynthetically generated ATP to the cytosol. Additionally, our model predicts that the metabolites imported into the companion cell are not necessarily the same metabolites that are exported in phloem sap; phloem loading is more efficient if certain amino acids are synthesized in the phloem tissue. Surprisingly, in our model predictions, the proton-pumping pyrophosphatase (H^+^-PP_i_ase) is a more efficient contributor to the energization of the companion cell plasma membrane than the H^+^-ATPase.

## Introduction

The transport of sugars and other key nutrients such as amino acids from source leaves via the phloem to sink tissues is essential for the growth of plants (van Bel 2021). While xylem works through the capillary action of water and dissolved ions along lifeless conduits (Strasburger 1891; Bollard 1960), phloem tissue is composed of living, tube-like sieve elements (van Bel 2003). Although sieve elements are living cells, they are generally considered almost metabolically inert whose primary purpose is as conductors of phloem sap (Esau 1939; van Bel 2003). Their symplasts are connected at each end by sieve plates which allow fluid flow and the passage of molecules such as sugars and amino acids [with size limits up to at least 67 kD (Stadler et al. 2005; van Bel 2018)]. This flow is vulnerable to structural breaches, for example, by herbivory, and this triggers a blockage via callose (Esau et al. 1953; van Bel 2006; Barratt et al. 2011).

To facilitate their role as living pipes, sieve elements contain a stripped back cellular machinery. They are devoid of a nucleus, functioning photosynthetic chloroplasts [although they do contain other plastids capable of storing starch (Behnke 1991)], and vacuoles (van Bel et al. 2002). In addition, their mitochondria are relatively rudimentary and, while metabolically active (McGivern 1957; Lee et al. 1971; Moninger et al. 1993), are thought unlikely to have the same capabilities as those in other cell types (Esau et al. 1953; Esau and Cronshaw 1968; Behnke et al. 1990; Cayla et al. 2015). The roles of these organelles are taken up by adjacent companion cells. Companion cells are symplastically linked to adjacent sieve elements so that metabolites can flow freely between them (Goodwin 1983; Stadler et al. 2005). Necessary mRNA transcripts are produced in companion cells for use in sieve elements (van Bel 1996; Ayre et al. 2003; Cayla et al. 2015) as well as small (<67 kD) proteins (Stadler et al. 2005).

It is well established that osmotic currents drive the flow of sap through phloem (Münch 1930; De Schepper et al. 2013; Knoblauch et al. 2016). This means that the energy cost of phloem transport is primarily in the maintenance of the concentration gradient driving these osmotic currents. In the source leaves of Arabidopsis (*Arabidopsis thaliana*) and similar herbaceous angiosperms, companion cells play an important role in actively importing photoassimilates produced in the mesophyll into the phloem tissue to maintain this gradient (Otero and Helariutta 2017). Sugars and amino acids produced in the leaf are passively exported from mesophyll cells into the apoplast. The bioenergetic force behind the loading of sugars and amino acids from the apoplast into companion cells is generated by proton gradients at the companion cell plasma membrane (Taiz 2010). The proton gradient across the plasma membrane is maintained by actively pumping protons from the companion cell cytosol to the apoplast. This allows companion cells to use proton-coupled plasma membrane symporters to import the photoassimilates in the apoplast against their concentration gradients but down the proton concentration gradient (Haritatos et al. 2000; Taiz 2010).

The proton motive force (PMF) is created and maintained by proton-pumping ATPases (H^+^-ATPases) and proton-pumping pyrophosphatases (H^+^-PP_i_ases) (VanBel 1995; Zhen et al. 1997; Langhans et al. 2001; Hafke et al. 2005). Both have been localized to companion cell membranes (DeWitt and Sussman 1995; Paez-Valencia et al. 2011; Pizzio et al. 2015). These molecular pumps can harness the energy released from hydrolyzing ATP or PP_i_ to pump protons out of the cell against the concentration gradient. It is unclear to what extent the generation of the companion cell membrane PMF is split between these 2 pumps. Companion cell–specific overexpression of H^+^-PP_i_ase increases plant growth (Khadilkar et al. 2016), and knockdown produces dwarf plants (Primo et al. 2019). Similarly, hyperactivation of H^+^-ATPase may also increase plant growth under low pH conditions (Robertson et al. 2004), while knockout of the companion cell–specific H^+^-ATPase in *Nicotiana plumbaginifolia* also results in dwarf plants (Zhao et al. 2000). However, in Arabidopsis, homozygous knockout of the companion cell–specific H^+^-ATPase was not achieved due to its potential role in pollen development (Robertson et al. 2004). These results suggest that the capacity of the proton pumps is potentially a limiting factor for phloem transport and plant growth. They also demonstrate some redundancy in the role of the 2 proton pumps in the maintenance of the companion cell PMF, perhaps making it more robust to internal substrate availability, i.e. ATP or PP_i_.

There has been speculation that, given the presence of 2 pumps and the thermodynamics involved, the plasma membrane H^+^-PP_i_ases may actually be working in reverse—using the PMF to synthesize PP_i_ (Davies 1997; Rocha Façanha and de Meis 1998; Gaxiola et al. 2016; Khadilkar et al. 2016; Scholz-Starke et al. 2019). One reason put forward for generation of PPi in this way is that it could be used as a metabolic energy source to reduce the ATP cost involved in sucrose degradation (Sonnewald 1992; Gaxiola et al. 2012; Gutiérrez-Luna et al. 2018; Igamberdiev and Kleczkowski 2021) allowing companion cells to maximize ATP generation through respiration of sucrose. The high ATP consumption required to maintain companion cell PMF and the high sucrose concentrations in companion cells underpin the arguments for this alternative sucrose degradation pathway. Most persuasively, transcript abundance of the sucrose-proton symporter 1 (*SUC1*), encoding a sucrose-proton symporter, was found to increase in *Arabidopsis* plants overexpressing H^+^-PP_i_ase (Gonzalez et al. 2010; Gaxiola et al. 2012).

Because PP_i_ is generated from a variety of biosynthetic reactions including synthesis of ribonucleic acids and protein, a full understanding of the respective contributions of the plasma membrane H^+^-ATPase and H^+^-PP_i_ase pumps to companion cell energetics can only be reached after a comprehensive analysis of fluxes throughout the companion cell metabolic network. Recently, 2 Arabidopsis leaf cell–specific transcriptome studies were published that were able to provide some clues as to metabolic function in the companion cell (You et al. 2019; Kim et al. 2021). However, it is difficult to say anything definitive about enzyme concentration, let alone reaction flux, solely from transcriptome data (Lan et al. 2012; Schwender et al. 2014; Peng et al. 2018). We therefore decided to integrate the published Arabidopsis cell–specific transcriptome data with a leaf-scale flux balance analysis (FBA) model of central metabolism that incorporates the metabolism of the mesophyll, companion cells, and phloem sieve elements, the goal being to resolve uncertainties about the metabolism of companion cells and sieve elements.

Flux balance analysis models are mathematical models that represent cellular metabolism as a matrix of stoichiometric equations of the relevant metabolic reactions. System inputs (e.g. carbon fixation rates, incident light intensity) and outputs (e.g. biomass accumulation rate, phloem transport rate) are included in the matrix, and constraints on the flux through relevant reactions are added based on rates of biosynthesis of cell structures and biomass polymers and the biophysics inherent in the system (e.g. charge balancing, diffusion limits). The range of reaction flux values that, when multiplied by the stoichiometric matrix, return a net zero change for every metabolite (representing metabolic steady state) then represents the range of fluxes that each reaction can plausibly take while preserving conservation of mass. This range of solutions can then be analyzed to determine properties of the system that may not otherwise have been evident. Because the feasible solution space of this set of fluxes is large, an optimization goal is included as an “objective function” to help home in on biologically relevant fluxes. Previous leaf FBA models which maximized biomass accumulation or phloem transport rate, while at the same time minimizing the sum of fluxes (a proxy for the efficiency of the network in terms of the total amount of enzymes required), as their objective function have been able to give insight into cell metabolism (Sweetlove and Ratcliffe 2011; Cheung et al. 2014, 2015; Nikoloski et al. 2015; Shameer et al. 2019). Integrating transcriptome data into such models using carefully considered algorithms that look at transcriptome data across all metabolic pathways covered by the model not only reduces the potential solution space of the model but can act as a weighting so that enzymes with high transcript abundance are more likely to carry high flux in the chosen model solution (Robaina Estévez and Nikoloski 2014; Robaina-Estévez et al. 2017; Scheunemann et al. 2018). Here, we describe the results of detailed analysis of this model and discuss the implications of our findings for the efficiency and metabolic underpinnings of phloem loading.

## Results

### A tissue-scale model of metabolism of an Arabidopsis source leaf incorporating phloem loading

To develop a tissue-scale model of phloem metabolism, we started with a diel FBA model of central plant cell metabolism in the leaf (Shameer et al. 2018, 2019) and adapted it to describe the cells involved in phloem loading (defined as the import of metabolites into the companion cell–sieve element complex). We determined the most necessary of these to be mesophyll cells, companion cells, and sieve elements—the cells most implicated in the synthesis, loading, and long-distance transport of the photoassimilate component of phloem sap, respectively (van Bel 1996; Haritatos et al. 2000). We included sieve elements in the leaf and sieve elements in the petiole as 2 distinct cell types in an effort to capture the continuous cost of sucrose retrieval and cell maintenance away from the energy-rich source leaf.

**Figure 1.**
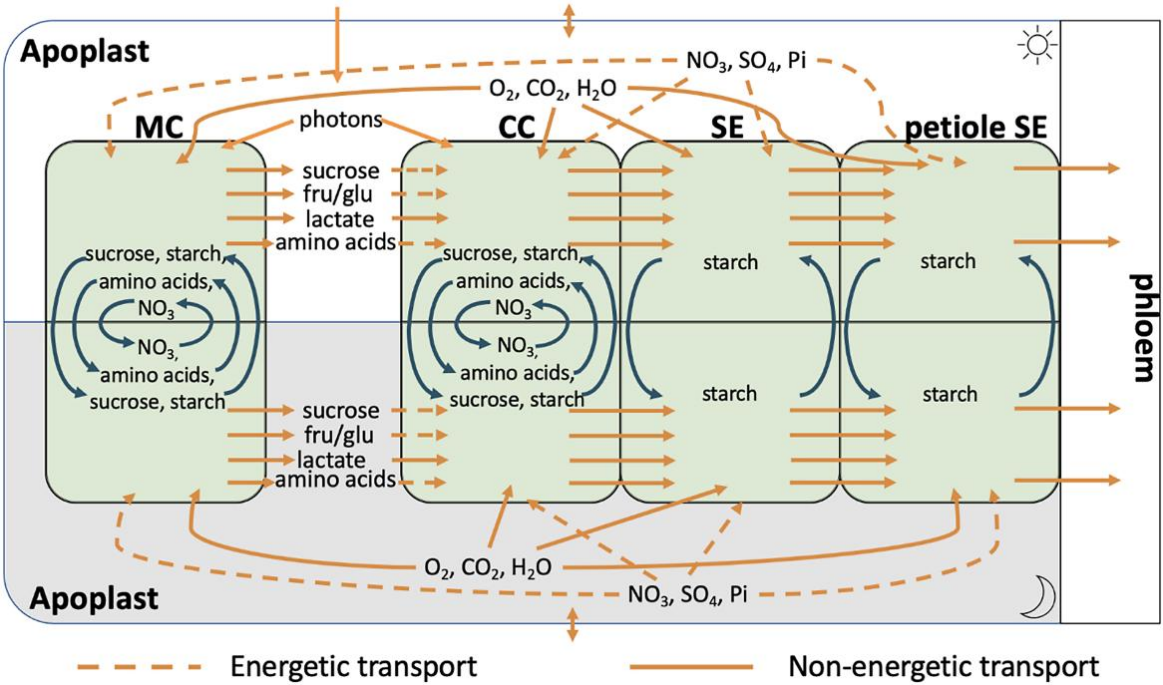
Schematic of the diel–multicell–FBA model of leaf tissue showing the inputs, outputs, and exchanges of metabolites, gases, and minerals between the 4 cell types considered. Each cell contains a core stoichiometric model of central metabolism, and fluxes are scaled to account for the different proportions of each cell type in the leaf. Each cell model is further replicated to account for the light phase (above) and the dark phase (below) with certain metabolites and minerals allowed to be passed between the phases as indicated. Since sieve elements do not have vacuoles, only starch is allowed to accumulate in them. The cells are all connected via the apoplast (this is also split into light phase apoplast and dark phase apoplast). However, only the companion cell, sieve element, and petiole sieve element cells are symplastically connected; sugars and amino acids must transit through the apoplast in order to move between the mesophyll and companion cells. CC, companion cell; MC, mesophyll cell; SE, sieve element (in the leaf); petiole SE, sieve element in the petiole, fru, fructose, glu, glucose.

A schematic summary of the model is shown in Fig. 1. Each cell type was modeled as a distinct diel FBA cell—composed of 2 metabolic models one each for the light phase and the dark phase with these 2 phase models connected by accumulation reactions that mimic the build-up of starch, sucrose, nitrate, malate, and amino acids during either the light or dark phase for use in the opposite phase (Cheung et al. 2014). The difference between the light phase and dark phase models is the flux of incident photons which is only present in the light phase model. Such models were constructed for each of the 4 cell types being considered. The light phase cell models were connected via a light phase apoplast compartment and the dark phase cells similarly connected via a dark phase apoplast compartment. Companion cells and sieve elements were connected via the symplast using no-cost “linker” reactions that allowed free exchange of the majority of model metabolites. We excluded large molecules from this symplastic transport, such as heteroglycans, polysaccharides (e.g. cellulose and starch), and lipids as well as superoxide, protons, and iron ions from these transport reactions based on the size limit in companion cell–sieve element plasmodesmata and the unrealistically high transport fluxes we initially saw transporting charge between cells. Nucleotides were only allowed to travel from companion cells to sieve elements but not from sieve elements to companion cells to prevent similarly unlikely energy transport between cells. Without these exclusions, the model transported substantial quantities of ATP from companion cells to sieve elements via energy-rich lipids or directly as ATP or equivalent nucleotide triphosphates. The petiolar sieve element component of the model included a phloem transport rate reaction that carried photoassimilates to the bulk phloem in the ratios measured by Wilkinson and Douglas (2003). The flux through this reaction represents the bulk flow rate of metabolites (sucrose and amino acids) in the phloem. The dark phase phloem transport rate was constrained to a third of the light phase phloem transport rate. Upper-bound constraints were placed on all fluxes in each phloem cell type to reflect the relative quantity of these cells relative to mesophyll cells in a mature Arabidopsis leaf (see “Materials and methods”).

Our model was further constrained by limiting the ribulose bisphosphate carboxylase–oxygenase flux in mesophyll cells so that the total model carbon assimilation rate matched the rate measured by Donahue et al. (1997). Cell maintenance costs were estimated for mesophyll cells based on the incident light as implemented by Töpfer et al. (2020). Maintenance costs for other cell types were then determined based on comparative cell numbers. Protein and mRNA turnover costs were also estimated based on experimental measurements (Piques et al. 2009; Li et al. 2017; Sorenson et al. 2018; Szabo et al. 2020) and imposed as model constraints. A sucrose leak and a sucrose retrieval flux were added to petiolar sieve elements based on data from Gould et al. (2012). All vacuole metabolic reactions were removed from sieve elements, including vacuolar metabolite accumulation. Due to the observed presence of starch-containing plastids (Behnke 1991), starch accumulation was allowed in sieve element plastids although the reaction carried no flux in our model solutions. Transport of amino acids through the apoplast was constrained to only flow in the direction of mesophyll to companion cell.

We used the cell type–specific transcriptome data collected by Kim et al. (2021) from 6-wk-old *Arabidopsis* plants to weight the flux solution using a variation of the RegrEx algorithm (Robaina Estévez and Nikoloski 2015). The original RegrEx algorithm uses linear regression to find a model solution in which fluxes are the closest quantitative match transcripts encoding the respective enzymes. RegrEx has since been used to model multicellular tissues (Robaina-Estévez et al. 2017; Scheunemann et al. 2018) relying solely on transcript abundance data sets to define flux in the cell types. However, since it is well known that transcript abundance and fluxes can be rather divergent, we modified the RegrEx algorithm to make it a hierarchical optimization problem in which the algorithm first looks for solutions that minimize difference to transcripts and then looks for solutions within that space that minimize the sum of fluxes of the model (as a proxy for efficient use of enzyme capacity). Since there are a large number of reactions in our model without corresponding transcripts in the transcriptome data set, greater weight is given to the first objective as the sum of fluxes is a much larger quantity than the sum of differences between fluxes and transcripts. Effectively, we use the transcriptome data to skew the solution space then also minimize the sum of fluxes so that pathways containing reactions with high mRNA abundance will be more likely to have a high flux and pathways containing reactions with low mRNA abundance will be more likely to have a low or zero flux.

The RegrEx algorithm was also modified by scaling the normalized transcriptome data to match the cell ratios in our model. In the case of companion cell and sieve element reactions where companion cells synthesize the transcripts for both cell types, the objective was to minimize the difference between the sum of fluxes in companion cells and both sieve elements and their corresponding transcript abundance in companion cells. Previous analysis of transcriptome and metabolic flux in plants suggested an overall correspondence between changes in transcript abundance and changes in flux of 32% (Schwender et al. 2014). To ensure that the minimization of flux objective did not result in zero phloem loading, the transcriptome-weighted model solution was constrained so that the difference between its phloem transport rate and that of a parsimonious solution (see “Materials and methods” for more detail) was no greater than 32%. The model was then solved as a single optimization problem that included each cell type and both temporal phases (day and night). To ensure the robustness of model predictions, we also performed flux variability analysis (FVA) on the transcriptome-weighted model to determine the ranges the flux through each reaction could take while maintaining the shortest distance from (but not necessarily matching) the transcriptome data. Our model suggests the likely distributions of ATP generation and consumption, as well as likely metabolic activity in companion cells and sieve elements. For full details, see the “Materials and methods” section.

The addition of phloem cells to the diel leaf model does not have a pronounced effect on the metabolic behavior of mesophyll cells with flux distributions (see Supplemental Table S1; Supplemental Fig. S1) broadly similar to those reported previously (Cheung et al. 2014, 2015; Shameer et al. 2019). These included the requirement for substantial mitochondrial ATP synthesis in the mesophyll in the light phase as well as export of reducing equivalents from the chloroplast via the malate valve (Shameer et al. 2019). The broad patterns of metabolite accumulation in day or night were also similar to previous reports (Cheung et al. 2013, 2014, 2015).

### Companion cell photosynthesis functions in the model to generate reducing power and ATP but with no carbon fixation

One of the most notable features of the model solution was the unusual metabolism in companion cell chloroplasts. We modeled companion cell chloroplasts as metabolically identical to mesophyll cell chloroplasts, i.e. the same reactions were allowed. However, we imposed a constraint on the maximum rate of photosynthesis in companion cell chloroplasts reflecting the comparative light-capturing capacity of mesophyll and companion cells due to the different proportions of each cell per unit leaf and the significantly reduced total chloroplast volume in companion cells (Paramonova et al. 2002; Cayla et al. 2015). These amount to restrictions on companion cell photon capture (Photon_ep) to 20% of the upper-bound–constraining mesophyll cell photon capture on a per cell basis (Supplemental Table S1).

With these constraints, companion cell chloroplasts were predicted by the model to still generate approximately half the amount of ATP as mesophyll cell chloroplasts on a per cell basis. This can be seen in the predictions for the plastidial ATP synthase flux in each cell when scaled by the number of each cell type per unit leaf area (Supplemental Fig. S2). This effectively modifies the in silico leaf to contain equal proportions of mesophyll and companion cells rather than the 20:1 ratio we have used in the model (see “Materials and methods”) leading to ATP biosynthesis estimates for the 2 cell types on an equivalent per cell basis. This leads to 12.9 and 20.5 *µ*mol ATP generated per m^2^ leaf area per s for companion cell and mesophyll chloroplasts, respectively. Flux variability analysis shows the amount of ATP produced by the companion cell chloroplast to be consistent across all optimal solutions (Supplemental Table S1). The companion cell chloroplast met 67% of the total light phase ATP needs of the companion cells. Of the remaining ATP needs, 19% was provided by mitochondrial ATP synthase, and the rest was produced in glycolysis or biosynthesis reactions. In the dark phase, mitochondrial ATP synthase contributed ∼84% of the companion cell's ATP needs (calculated by dividing the flux through each ATP synthase reaction by the combined flux of all reactions that synthesize ATP in the companion cells during the light and dark phases independently; see Supplemental Fig. S2 and Supplemental Table S1 for specific flux values). Over the day/night period, companion cell chloroplasts produced 14% more ATP than companion cell mitochondria.

The high ATP productivity of companion cell chloroplasts in the model, despite them absorbing a small proportion of total intercepted light, was the result of a greater fraction of the light absorbed by companion cells being used for photochemistry—a low amount of nonphotochemical quenching compared to the substantial amount at the whole leaf level at the light intensity we used (Müller et al. 2001; Li et al. 2009). It also implies that the companion cell could potentially have an energy-limited, rather than carbon-limited, metabolism.

**Figure 2.**
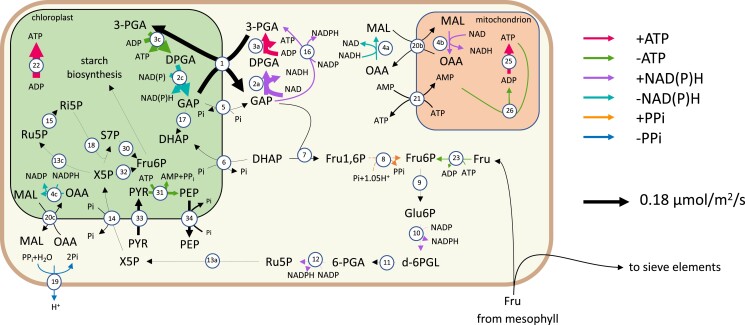
A flux map of the principal reactions involved in companion cells during the light phase. Arrow thickness indicates reaction flux value. The key on the right explains the colours used to indicate the production/consumption of key metabolites. The reactions shown are 1, GAP-3–PGA shuttle; 2, (NADP) GAP dehydrogenase; 3, 3-PGA kinase; 4, malate dehydrogenase; 5, GAP–Pi shuttle; 6, DHAP–Pi shuttle; 7, FBP aldolase; 8, FBP phosphotransferase; 9, Glu6P isomerase; 10, Glu6P dehydrogenase; 11, 6-phosphogluconolactonase; 12, gluconate-6-phosphate dehydrogenase; 13, Ru5P epimerase; 14, X5P–phosphate shuttle; 15, ribose-5-phosphate isomerase; 16, 3-PGA dehydrogenase; 17, triosephosphate isomerase; 18, transketolase; 19, H^+^-pyrophosphatase; 20, malate–oxaloacetic acid shuttle; 21, ATP–AMP shuttle; 22, plastidial ATP synthase; 23, fructose kinase; 25, mitochondrial ATP synthase; 26, adenylate kinase; 30, transaldolase; 31, pyruvate orthophosphate dikinase; 32, transketolase; 33, pyruvate channel; 34, phosphoenol pyruvate channel; and 35, plastidial ATP synthase. The metabolites shown are GAP, glyceraldehyde-3-phosphate; DPGA, 3-phospho-d-glyceroyl phosphate; 3-PGA, 3-phosphoglycerate; DHAP, Dihydroxy acetone phosphate; MAL, malate; OAA, oxaloacetic acid; Glu1P, glucose-1-phosphate; Glu6P, glucose-6-phosphate; d-6PGL, 6-phosphoglucono-∂-lactone; 6-PGA, 6-phosphogluconate; Ru5P, ribulose-5-phosphate; X5P, xylulose-5-phosphate; and Ri5P, ribose-5-phosphate.

From the predicted fluxes of chloroplast ATP consuming reactions (see A), we can see that the majority of companion cell photosynthesis-derived energy (51%) was predicted to be used in amino acid biosynthesis pathways in the chloroplast, while the rest was exported to the cytosol using either the glyceraldehyde 3-phosphate–3-phosphoglycerate shuttle or the pyruvate–phospho*enol*pyruvate shuttle (Fig. 2) to maintain the plasma membrane proton gradient necessary to continue sucrose and amino acid import. No carbon fixation by Rubisco was predicted to occur in companion cell chloroplasts in the model. In contrast, in mesophyll chloroplasts, the model predicted that 80% of photosynthetically generated ATP is used to fix carbon leaving the other 20% for use in other cellular processes.

A study of chloroplast structure (Paramonova et al. 2002) showed that companion cell chloroplasts have less pronounced grana than mesophyll cell chloroplasts. This could imply reduced or nonexistent function of photosystem II (Dekker et al. 2002; Dekker and Boekema 2005) in these cells. We analyzed the effect that removal of companion cell photosystem II had on the model solution and found that, while this did reduce the total amount of ATP available in companion cells (by 33%—calculated as the total amount of ATP synthesized in companion cells), companion cell photosynthesis still provided a substantial proportion of ATP used in the companion cell (45% versus 59% of the combined ATP generated over the course of the day and night cycle when photosystem II was present) and there was still no carbon fixation (A, B). The lack of photosystem II meant that thylakoid plastoquinone needed to be reduced via the ferredoxin–plastoquinone reductase pathway and this consumed all of the electrons released by photosystem I eliminating NADPH synthesis. The NAD(P)H requirements of the Calvin–Benson cycle were fulfiled by importing reducing power from the cytosol via the malate–oxaloacetic acid shuttle and as a by-product of the serine and histidine biosynthesis pathways. The lack of reducing power in companion cells greatly reduced mitochondrial ATP synthase activity as well (by 30.6% during the light phase) (Supplemental Table S1). However, the companion cell chloroplast was still capable of generating enough ATP to minimize the need for catabolism of metabolites imported into the companion cell (Supplemental Table S1).

### The transcriptome-weighted model indicates that H^+^-PP_i_ase is the more efficient plasma membrane proton pump in companion cells

There is evidence of both Arabidopsis H^+^-ATPase 3 (AHA) and type 1 Arabidopsis H^+^-PP_i_ase (AVP1) pumps on the plasma membrane of companion cells (Paez-Valencia et al. 2011; Gaxiola et al. 2016; Otero and Helariutta 2017). Given the presence of both proteins, it could be that both pumps are in use and important in maintaining the companion cell plasma membrane proton motive force (PMF), which is necessary for phloem loading. However, there is some debate about whether H^+^-PP_i_ase contributes to or consumes the PMF in companion cells (Davies 1997; Langhans et al. 2001; Gaxiola et al. 2012; Segami et al. 2018).

In our transcriptome-weighted model solution, only the H^+^-PP_i_ase carried flux (see Supplemental Table S1). The transcript abundance for several pyrophosphate-synthesizing reactions weights our model toward a solution in which sufficient PP_i_ is generated within the companion cells for H^+^-PP_i_ase to maintain the majority of the PMF required for uptake of sugars and amino acids. While there was no direct information for H^+^-PP_i_ase transcript abundance in the published data set, pyrophosphate produced in protein/RNA turnover reactions and by pyrophosphate–fructose-6-phosphate phosphotransferase in the transcriptome-weighted model solution was sufficient for the H^+^-PP_i_ase pump to maintain the companion cell's PMF with the modeled photoassimilate import. Pyrophosphate production requires additional enzymes to catalyze the necessary reactions, and these consume ATP which would otherwise directly power the H^+^-ATPase pump. Flux variability analysis of the transcriptome-weighted model solution indicated that there are several viable pyrophosphate-producing pathways such as those that involve GDP-glucose pyrophosphorylase and pyruvate orthophosphate dikinase. Many of these pathways are involved in polysaccharide synthesis, and the transcriptome abundance of the encoded enzymes in several of them suggests there may be some companion cell polysaccharide synthesis, such as in cell wall maintenance (Wolf et al. 2012) and callose formation in sieve elements. There is evidence of dynamic callose formation and degradation at phloem plasmodesmata and, potentially, sieve plates, to control flow (Barratt et al. 2011; Vatén et al. 2011; Xie and Hong 2011). The production of either of these carbohydrates would result in a net conversion of ATP to PP_i_ which can then also be used to pump protons out of the cell. Additionally, due to the amount of PP_i_ produced by protein and mRNA turnover, FVA showed there was always a role for H^+^-PP_i_ase.

To explore this further in silico, we re-ran the model limiting H^+^-PP_i_ase to 10% of its previous flux. This results in the H^+^-ATPase pump taking over as the dominant plasma membrane proton pump in companion cells (pumping 7.8 times more protons than H^+^-PP_i_ase) and an increase of 7% in the flux through cytosolic inorganic pyrophosphatase (Supplemental Table S2). Notably, it also reduces the rate of phloem transport by ∼3%, confirming that utilization of H^+^-PP_i_ase to energize the companion cell plasma membrane is a stoichiometrically more efficient solution. Furthermore, when we investigated an optimized model (parsimonious FBA) without any transcriptome weighting, H^+^-PP_i_ase was still the preferred pump due to the pyrophosphate synthesized in protein and mRNA turnover (Supplemental Table S2, Supplemental Fig. S1). However, complete reliance on H^+^-PP_i_ase which our model predicts is possible is contradicted by the results of H^+^-ATPase knockout (Zhao et al. 2000; Haruta et al. 2010). One explanation for this is that the in vivo capacity of the H^+^-PP_i_ase proton pump is considerably lower than that specified in our model where the upper bounds for flux through both H^+^-ATPase and H^+^-PP_i_ase are due solely to cell ratio constraints and do not take into account the actual abundance and kinetics of these proteins. It is likely that the abundance of both proton pumps is a limiting factor in the flux through these reactions (Robertson et al. 2004; Khadilkar et al. 2016). Despite this strong model preference for H^+^-PP_i_ase, there is a role for both pumps in the transcriptome-weighted model solution (as shown in the FVA results, Supplemental Table S1) and it is likely that they both contribute to the maintenance of the companion cell PMF in vivo with compensatory roles under different types of stress. Nevertheless, our model emphasizes the potential importance of the H^+^-PP_i_ase for phloem loading given PP_i_ production by metabolism within the companion cells.

### Uptake of metabolites from the apoplast into the companion cell

**Figure 3.**
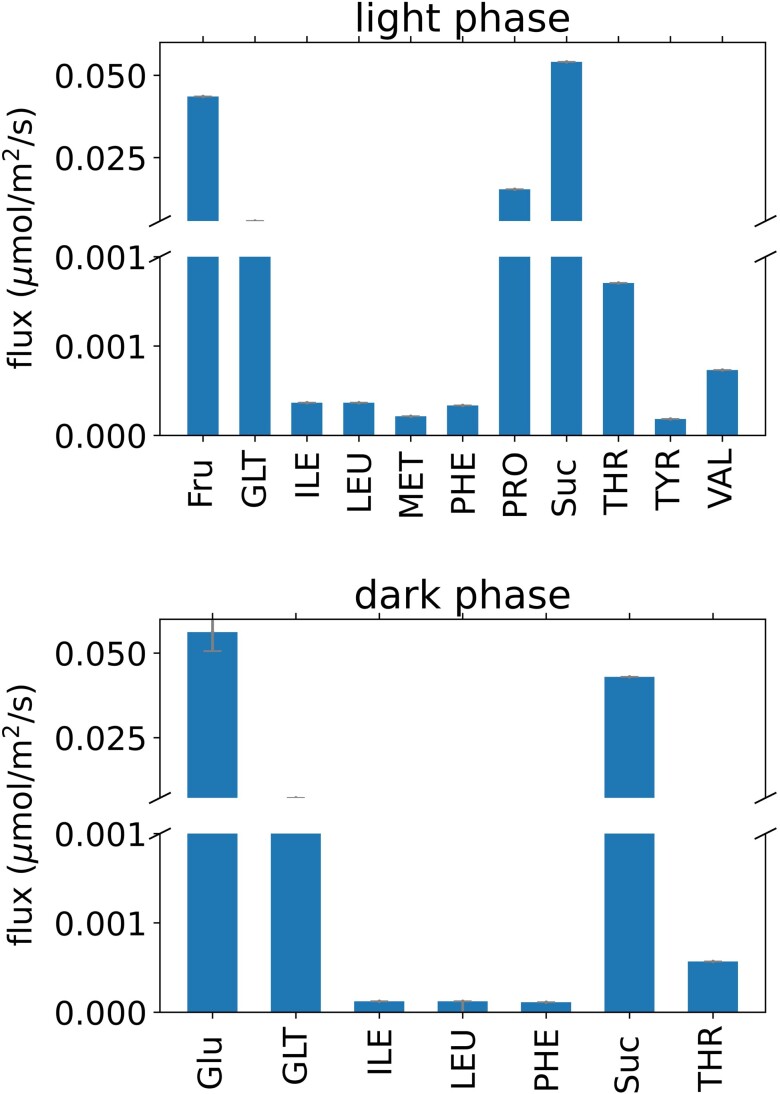
Predicted fluxes of metabolite transport via the apoplast between mesophyll and companion cells in the transcriptome-weighted model solution. Abbreviations are as in Fig. 2.

As expected, sucrose import is the largest flux (0.054 and 0.043 *µ*mol m^−2^ s^−1^ in the dark and light phases, respectively) of the metabolites actively transported into the companion cell from the apoplast (Fig. 3). Unexpectedly, there was a similarly high fructose import flux (0.044 *µ*mol m^−2^ s^−1^ in the light phase) which was then used in glycolysis. On closer inspection, exporting fructose rather than sucrose allowed the mesophyll cells to generate more pyrophosphate to power the vacuolar H^+^-PP_i_ase pump for storage of metabolites in the vacuole between day and night phases. The model predicted that the remaining energetic demands of the companion cells were largely met by companion cell photosynthesis and catabolism of imported amino acids. Approximately half of the imported sucrose (46%) was passed unchanged via the symplast into the phloem. The remaining portion (54%) was partially consumed, not within companion cells, but within the sieve elements to provide energy for cell maintenance and carbon skeletons for amino acid synthesis. Amino acid synthesis from the products of partial sucrose catabolism resulted in ∼70% of the modeled carbon fixed by mesophyll cell Rubisco being exported in the phloem sap. Sucrose was catabolized in sieve elements either by sucrose synthase or invertase. This is consistent with evidence of sieve element–specific sucrose synthase isoforms (Barratt et al. 2009; Yao et al. 2020) and the presence of both enzymes in the *Nicotiana tabacum* sieve element proteome (Liu et al. 2022).

**Figure 4.**
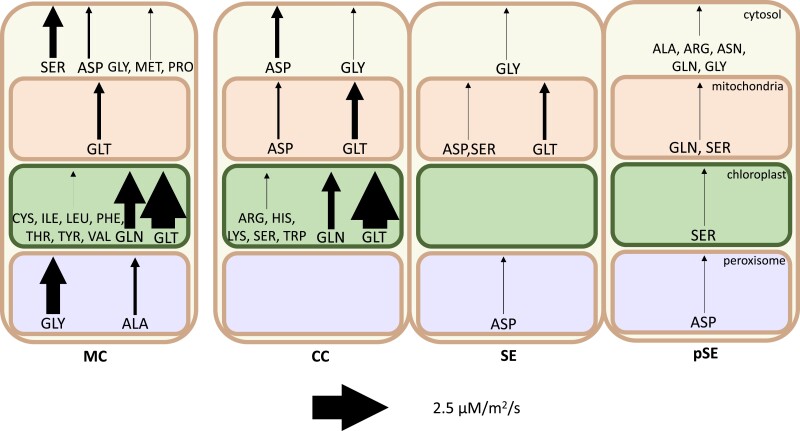
Amino acid synthesis by compartment in each cell in the light phase. Arrow thickness indicates magnitude of synthesis flux. Note that flux is scaled by cell ratios to better compare the activity between individual cells. MC, mesophyll cell; CC, companion cell; SE, sieve element; pSE, petiolar sieve element.

Of the amino acids that were predicted to be taken up by the companion cell (Fig. 3), most were transported unchanged through the connected symplast of companion cells and sieve elements and into the bulk phloem. The exceptions to this were proline, glutamate, and, in the light phase, aspartate and arginine. All 4 of these were used in the model to synthesize the remaining amino acids within phloem cells (Fig. 4) and to provide additional ATP and reducing power (Supplemental Table S1). While it is not exported in phloem sap, the model predicts that proline is also among the metabolites taken up by the companion cells from the apoplast. The extra reducing power obtained from converting proline into glutamate in phloem tissue via mitochondrial proline dehydrogenase and 1-pyrroline-5-carboxylate dehydrogenase allows the mesophyll cells to export fractionally more reducing power to the phloem. If this pathway was blocked in the model, proline is replaced with greater arginine and glutamate export to phloem cells and results in a small decrease in the maximum phloem transport rate of the model.

### Amino acid biosynthesis could occur in phloem tissue

Many of the amino acids found in Arabidopsis phloem sap were imported into companion cells from the apoplast (and hence biosynthesized in the mesophyll) in our model solution. However, a large proportion were synthesized within the phloem tissue. Figure 4 depicts the relative amounts of amino acid biosynthesis occurring in each cell in our model solution during the light phase. In particular, our model suggests that arginine, asparagine, histidine, lysine, and tryptophan are more efficiently synthesized in phloem tissue from by-products of the oxidative pentose phosphate pathway and glycolysis than synthesized and transported from the mesophyll (see lack of range in FVA results, Supplemental Table S1).

This result arises partly from the transcriptome weighting and partly from the optimization objective of the model. Every cell in the model was predicted to carry flux through glycolysis and the oxidative pentose phosphate pathway to some extent to provide the ATP and NADPH necessary for cell maintenance. Of the 5 amino acids whose biosynthesis pathways branch off of these, our model predicted which would be most efficiently produced in the phloem, the main factor being the extent to which energy or reducing power was produced in their biosynthesis. This means that the histidine biosynthesis pathway which produces both NADH and pyrophosphate was active in companion cells in the model. Arginine, which is synthesized from aspartate, produces fumarate as a by-product, and this fumarate was used by the model to produce ATP in phloem tissue (companion cells and petiole sieve elements) via the TCA cycle. Asparagine, which can be synthesized from aspartate and ATP while producing pyrophosphate, was also predicted to be synthesized in the petiole sieve elements where the pyrophosphate was used by the model in sucrose catabolism.

Surprisingly, tryptophan and lysine were also predicted to be synthesized in companion cells in our model. For tryptophan, the explanation is that several reactions in the indole synthesis pathway have higher transcript abundance in companion cells than in mesophyll cells and the synthesis of tryptophan consumes this indole. The prediction of biosynthesis of lysine in companion cells is surprising because it consumes ATP and reducing power, and reactions in its synthesis pathway generally had higher transcript abundance in mesophyll cells (Supplemental Table S1).

In the dark phase when energy and reducing power are less abundant in mesophyll cells, and most other exported amino acids must be accumulated in the mesophyll to maintain phloem transport rate, our model predicted that the cheapest of these same “low-cost” amino acids will still be actively biosynthesized in the phloem tissue at night. While amino acids produced in phloem tissue are produced in both the light and dark phases, other than glutamate, our model predicts that all amino acids produced in the mesophyll for phloem transport rate must be produced during the light phase. They are then accumulated in the vacuoles for export in the dark phase.

Model solutions indicate that, apart from these and glycine, glutamine, alanine, aspartate, and serine which can be produced in either mesophyll or companion cells, the other amino acids are most efficiently produced in mesophyll cells. The full details of the ATP, NAD(P)H, and PP_i_ balancing in the model solution are shown in Supplemental Figs. S2 to S4.

### Adenylate kinase is necessary for model feasibility

**Figure 5.**
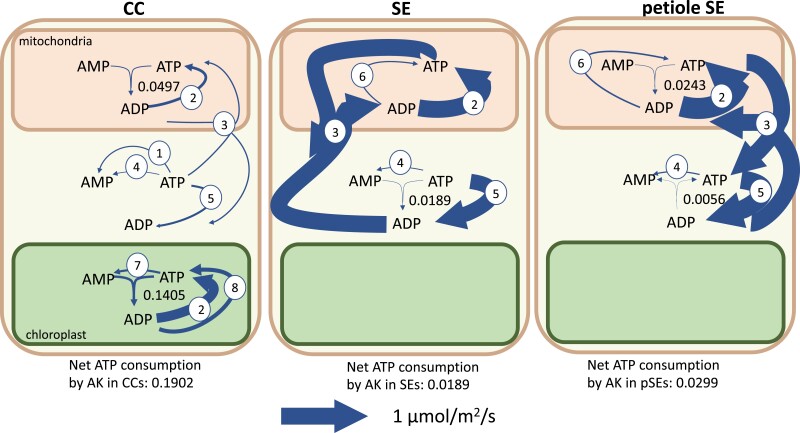
Flux map showing fluxes of adenylate kinases (AK) and related reactions in the transcriptome-weighted model solution. The net effect of adenylate kinase fluxes is a consumption of ATP. The reactions depicted other than adenylate kinase are 1, pyruvate kinase; 2, ATP synthase; 3, ATP–ADP exchanger; 4, protein/RNA turnover; 6, succinyl-coA synthetase; 7, pyruvate orthophosphate dikinase; and 8, phosphoglycerate kinase and acetyl-glutamate kinase. Arrow thickness is scaled to flux value as indicated. Other than adenylate kinase, only fluxes >0.05 *µ*mol m^−2^ s^−1^ are shown. The total ATP consumption by adenylate kinase in phloem cells is 0.2390 *µ*mol m^−2^ s^−1^. CC, companion cell; SE, sieve element. The ranges on the adenylate kinase fluxes are CC_m_, 0 to 0.1657; CC_c_, 0 to 0.1657; CC_p_, 0 to 0.1405; SE_m_, 0 to 0.1349; SE_c_, 0 to 0.1349; SE_p_, 0 to 0.1403; pSE_m_, 0 to 0.0299; pSE_c_, 0 to 0.0299; and pSE_p_, 0 to 0 *µ*mol m^−2^ s^−1^ ATP consumed where the subscripts m, c, and p indicate the mitochondrion, cytosol, and plastid, respectively.

Unexpectedly, we found that adenylate kinase (ATP + AMP -> 2ADP) flux is necessary for model feasibility and causes a net consumption of ATP in model solutions. Consistent with this, adenylate kinase mRNA has a relatively high abundance in the companion cell transcriptomes (Kim et al. 2021). Figure 5 shows the adenylate kinase fluxes and other reactions involving adenine nucleotides in the phloem cells. Despite the prediction of flux through the adenylate kinase reaction in all subcellular compartments in which it is present (mitochondrion, cytosol, and chloroplast), there was no net adenine nucleotide transport between the cytosol and chloroplast in any cell type of the model.

Systematic in silico knockout of each adenylate kinase in the model showed that mitochondrial and cytosolic adenylate kinase activity in phloem cells together have the greatest effect on the rate at which the model was able to export sugars and amino acids to the phloem. Knockout of these 2 reactions in either companion cells or sieve elements resulted in an infeasible model. The primary function of these adenylate kinase isoforms in the model was to rephosphorylate AMP produced in other cellular processes. In the model solutions, these were pyruvate orthophosphate dikinase for the chloroplast–cytosol phosphoenolpyruvate phosphate translocator, succinyl-coA biosynthesis in the TCA cycle, and protein/RNA turnover and resynthesis. Adenylate kinase activity in the mitochondrion allowed the model to balance the ratios of ATP, ADP, and AMP to match the organelle's adenine nucleotide exchangers for optimal ADP phosphorylation via mitochondrial ATP synthase. This activity occurs in all phloem cells modeled—companion cells, sieve elements, and petiolar sieve elements.

### Differences between the transcriptome-weighted model solution and an optimized parsimonious model solution

We compared the transcriptome-weighted model solution with a parsimonious model solution that was not influenced by the transcriptome data at all. This latter solution was calculated to maximize phloem transport rate and minimize model fluxes. The transcriptome-weighted solution and the parsimonious solution have the same objectives and constraints, but the transcriptome-weighted solution is allowed a lower phloem transport rate than the parsimonious solution so that it can also minimize the gap between the transcriptome data and model fluxes. This was allowed to give the model flexibility in case the transcriptome weighting led to less efficient metabolism overall. For example, the transcriptome data includes metabolic pathways such as cell wall and callose synthesis and fatty acid biosynthesis that would not be active in our mature leaf model unless a specific constraint was added. There is also the possibility that some reactions carry a different flux in vivo than in our model because of thermodynamic and kinetic considerations. And it may also be the case that less efficient pathways are utilized in vivo due to evolutionary reasons (i.e. evolution does not necessarily optimize). These differences between the transcriptome-weighted model and an optimized model may point toward potential targets for engineering more efficient phloem loading with consequent positive impacts on crop performance (Braun et al. 2014; Gaxiola et al. 2016; Pazhamala et al. 2020).

The majority of the differences between the 2 model solutions can be linked to the difference in phloem transport rate with most biosynthesis reactions running in the same cells, in the same ratios, but with reduced fluxes (Supplemental Table S1). With the lower energy and carbon needs in phloem tissue in the parsimonious solution, the starch and sucrose accumulation in the companion cell chloroplasts and vacuoles that the transcriptome-weighted solution predicts during the light phase for use in the dark phase is not necessary for optimal phloem transport rate.

There were also differences in amino acid metabolism with the parsimonious solution only importing threonine, isoleucine, methionine, and glutamate into the companion cells. The rest of the amino acids found in the phloem sap (and hence required in our model's phloem transport rate) were predicted to be synthesized within phloem cells (companion cells or sieve elements) in the parsimonious model. In general, there is an energetic or reducing power-based benefit in synthesizing these amino acids in phloem cells allowing companion cells to be more efficient in importing only the most energy-rich amino acids and synthesizing the rest from them. Several, such as glycine, glutamate, and glutamine, are just as easily produced in phloem tissue as mesophyll cells based on stoichiometry. The differences are based on the transcriptome data and could indicate potential engineering targets to increase the efficiency of phloem transport.

Additionally, fructose was not imported into the parsimonious model's companion cells. Companion cell chloroplasts in the parsimonious solution channeled all of the ATP and reducing power they produced into the GAP–3PGA export shuttle rather than the less efficient PEP–pyruvate shuttle. There was then enough ATP to satisfy cell maintenance costs, including protein and RNA turnover, and dephosphorylate fructose-1,6-bisphosphate to produce enough PP_I_ to import the required amino acids and sucrose. The less efficient PEP–pyruvate shuttle was used in the transcriptome-weighted solution due to a high transcript abundance of pyruvate kinase in all cell types. While pyruvate kinase is important in the glycolytic pathway (Ambasht and Kayastha 2002), its primary use in our model was in facilitating the PEP–pyruvate conversion.

## Discussion

### Chloroplast activity in companion cells

**Figure 6.**
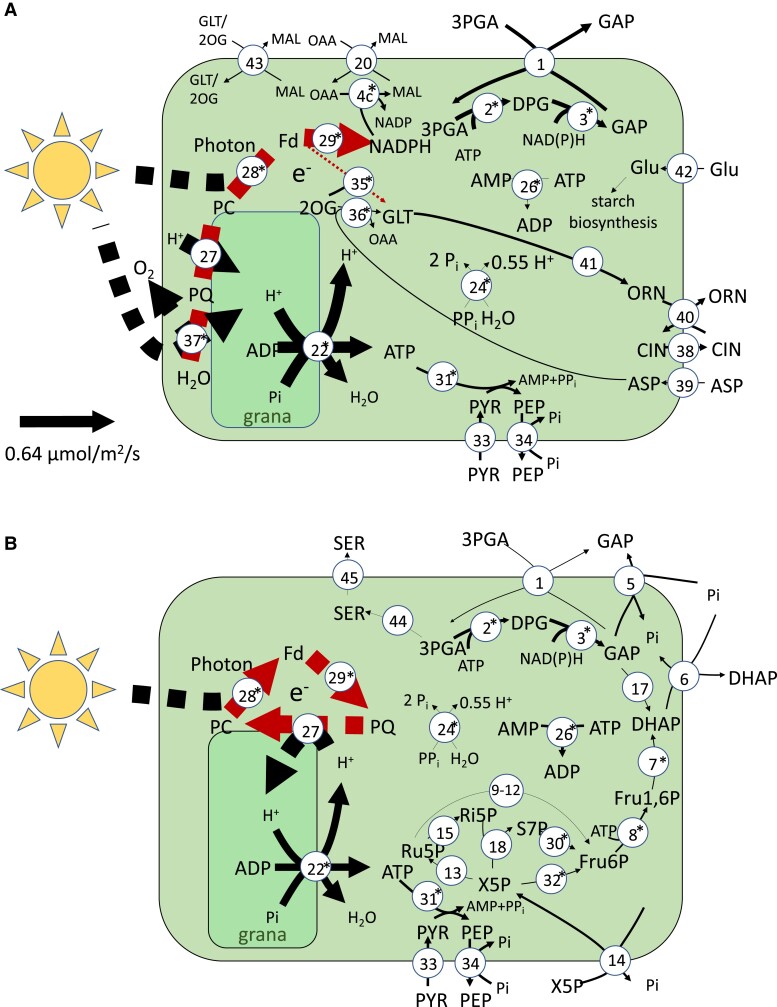
Energy metabolism in companion cell chloroplasts. **A)** A flux map of the transcript-guided model solution in the light phase companion cell chloroplast. The internal, dashed arrows indicate electron transport rather than mass flow. **B)** A flux map of the transcript-guided model solution in the light phase companion cell chloroplast when PSII is disabled. Asterisks next to reaction numbers indicate a lower transcript abundance in companion cells when compared with mesophyll cells. Reactions with flux less than 0.005 *µ*mol m^−2^ s^−1^ were excluded. The thickness of the lines of each solid arrow (excluding electron transport reactions) is scaled linearly to flux. The scale is shown on the left hand side of each diagram. The reactions shown are 1, GAP–3-PGA shuttle; 2, (NADP) GAP dehydrogenase; 3, 3-PGA kinase; 4, NADP^+^ malate dehydrogenase; 5, GAP–Pi shuttle; 6, DHAP–Pi shuttle; 7, FBP aldolase; 8, FBP phosphotransferase; 9, Glu6P isomerase; 10, Glu6P dehydrogenase; 11, 6-phosphogluconolactonase; 12, gluconate-6-phosphate dehydrogenase; 13, Ru5P epimerase; 14, X5P–phosphate shuttle; 15, Ri5P isomerase; 16, transketolase; 17, triosephosphate isomerase; 18, GDP kinase; 19, H^+^-pyrophosphatase; 20, malate–oxaloacetic acid shuttle; 21, ATP–AMP shuttle; 22, plastidial ATP synthase; 23, GDP-glucose pyrophosphorylase; 24, inorganic pyrophosphate; 26, adenylate kinase; 27, plastoquinol-plastocyanin reductase; 28, PSI; 29, ferredoxin–plastoquinone reductase; 30, transaldolase; 31, pyruvate orthophosphate dikinase; 32, transketolase; 33, pyruvate channel; 34, PEP channel; 35, glutamate synthase ferredoxin reaction; 36, aspartate aminotransferase; 37, PSII; 38, citrulline channel; 39, aspartate channel; 40, ornithine–citrulline shuttle; 41, ornithine synthesis pathway (from glutamate); 42, glucose channel; 43, malate–glutamate and malate–2-oxoglutarate shuttles; 44, serine biosynthesis pathway; and 45, serine channel. The metabolites shown are GAP, glyceraldehyde-3-phosphate; DPGA, 3-phospho-d-glyceroyl phosphate; 3-PGA, 3-phosphoglycerate; DHAP, dihydroxy acetone phosphate; MAL, malate; OAA, oxaloacetic acid; Glu1P, glucose-1-phosphate; GDP-Glu, GDP-glucose; Glu6P, glucose-6-phosphate; d-6PGL, 6-phosphoglucono-∂-lactone; 6-PGA, 6-phosphogluconate; Ru5P, ribulose-5-phosphate; X5P, xylulose-5-phosphate; Ri5P, ribose-5-phosphate; Fd, ferredoxin; PC, plastocyanin; PQ, plastoquinol; Glu, glucose; GLT, glutamate; GLN, glutamine; PYR, pyruvate; PEP, phosphoenol pyruvate; CLN, citrulline; ORN, ornithine; and SER, serine.

Our model analysis suggests that given the energy demand of phloem loading, photosynthesis in companion cell chloroplasts is required for the most efficient system. The bulk of the ATP and reducing power generated in the companion cell chloroplast was exported to the cytosol via metabolite shuttles, and there was no carbon fixation. Comparison of the structure of companion cell chloroplasts and mesophyll chloroplasts shows that those in companion cells have a thicker chloroplast peripheral reticulum with enlarged channels (Paramonova et al. 2002). This supports the likelihood of the high metabolite exchange between cytosol and chloroplast suggested in the model solution which transfers photosynthesis-derived ATP (i.e. the GAP–3PGA shuttle) and could be indicative of the different role companion cell chloroplasts play. While there is no significant difference between abundance of Rubisco transcripts in companion cells and mesophyll cells in the Kim et al. (2021) data set, several Calvin–Benson cycle enzymes and photosynthetic complexes do have significantly lower transcript abundance in companion cells compared with mesophyll cells (Fig. 6), including both photosystem I and II. In both the transcriptome-weighted and parsimonious model solutions, there was no flux through companion cell Rubisco in contrast to the relatively high expression. To test this, companion cell–specific knockdown of Rubisco would be required, which is beyond the scope of the current study. Alternatively, if the Calvin cycle is operational in companion cell chloroplasts and consuming ATP to fix carbon, then knockout of companion cell Rubisco would be a promising knockout target for improved efficiency of phloem loading as it would allow more of the photosynthetically generated ATP to be made available for this process.

**Figure 7.**
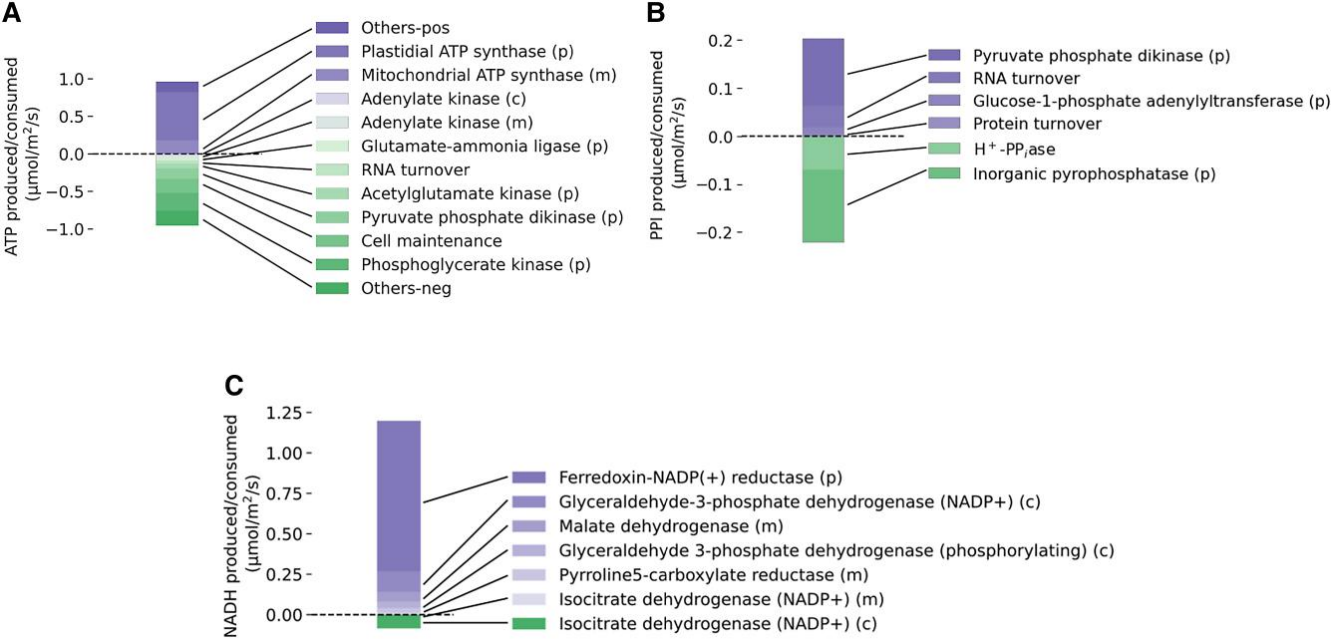
Synthesis and expenditure of energy and reducing power in companion cells during the light phase. **A)** A plot of the major reactions involved in ATP synthesis and consumption in companion cells during the light phase. **B)** A plot of the major reactions involved in PPI synthesis and consumption in companion cells during the light phase. **C)** A plot of the major reactions involved in NAD(P)H synthesis and consumption in companion cells during the light phase. (m), (p), and (c) indicate reaction occurs in the mitochondrion, chloroplast, and cytosol, respectively.

It is also interesting to note that in model solutions where flux through companion cell photosystem II was allowed, companion cell mitochondria were predicted to produce large amounts of ATP (Fig. 7) with the reducing power to facilitate this being derived from photosynthesis rather than from sucrose catabolism. The transfer of reducing power from chloroplast to mitochondrion is similar to that described by Bailleul et al. (2015) in diatoms but in our model was transferred via the chloroplast GAP–3PGA shuttle rather than via the malate valve as in diatoms. It is unclear what the respective capacities of these metabolite shuttles in companion cells are, and so this may be an unrealistically efficient flux pattern and could contribute to an overestimation of the capacity of the companion to generate. Should this be the case, it is likely that catabolism of sucrose would need to play a role as a source of ATP production in companion cells in contrast to the model prediction that no sucrose is catabolized in the companion cells. In this context, it is interesting to note that an Arabidopsis mutant completely deficient in all isoforms of sucrose synthase (Fünfgeld et al. 2022), including those expressed in companion cells, was able to grow and develop normally. However, this does not mean that no companion cell sucrose catabolism was occurring because invertase activity could take over the role in sucrose catabolism as it appears to in potato (*Solanum tuberosum* L.) sucrose synthase knockouts (Zrenner et al. 1995).

### H^+^-PP_i_ase as a pyrophosphate synthase

It has been proposed that the H^+^-PP_i_ase could work in reverse, exploiting the proton gradient either at the plasma membrane or tonoplast to allow pyrophosphate synthesis. Photoassimilate loading into companion cells is an energy-intensive process, and sucrose is necessarily highly concentrated in companion cells. Catabolizing a portion of the imported sucrose to generate the ATP required to import more of it is an effective solution to the high ATP cost of photoassimilate import into the phloem. In the sucrose catabolism pathway, several steps, namely, the conversion of UDP-glucose to glucose-1P and the phosphorylation of fructose-1P to fructose 1,6-bisphosphate, can be powered through either ATP or PP_i_ hydrolysis. Higher PP_i_ concentrations in the cytoplasm could increase the rate of these reactions, increasing the rate of sucrose catabolism and production of the ATP generated (Gaxiola et al. 2012, 2016; Schilling et al. 2017; Primo et al. 2019).

Additionally, it has been calculated that it is thermodynamically unlikely for a vacuolar H^+^-PP_i_ase protein to be hydrolyzing PP_i_ at 20 °C given available data on cytosolic ion concentrations and pH (Davies et al. 1993; Davies 1997). As the membrane potential difference is higher at the plasma membrane, the same arguments apply. While there is evidence of H^+^-PP_i_ase acting in both the PP_i_ hydrolyzing and synthesizing directions, its function at the plasmalemma of companion cells has yet to be unequivocally determined. It is quite possible that it is situation specific (Rocha Façanha and de Meis 1998; Seufferheld et al. 2004; Bao et al. 2009).

We do not see any PP_i_ synthesis via H^+^-PP_i_ase in any of our model solutions. In fact, due to the amount of PP_i_ produced from biosynthesis, our transcriptome-weighted phloem model indicates that H^+^-PP_i_ase is the dominant proton pump in *Arabidopsis* companion cells. Further, flux variability analysis indicates that the pump running in reverse, to synthesize PP_i_, would reduce phloem transport rate (Supplemental Table S1). This prediction can be traced to the constraint on carbon intake in mesophyll cells which limits the amount of sucrose they can produce and that companion cells can then import. Companion cells have smaller chloroplasts and the added ATP drain of importing photoassimilates so there is an energy bottleneck at the companion cell–apoplast interface.

### Transcriptome-weighted model predictions of metabolic activity in sieve elements predict surprisingly high rates of fructose catabolism

According to our model, 54% of the sucrose imported into the phloem tissue is partially consumed in the sieve elements to meet maintenance costs. Surprisingly, a similar amount of fructose (44% of the total amount of sucrose imported) is predicted to be imported for catabolism within phloem cells (Fig. 6). In addition, aspartate, glutamate, proline, and serine are also predicted to be catabolized in sieve elements to produce ATP or reducing power and other amino acids.

These predictions are consistent with the high transcript abundance of fructokinase and enzymes of glycolysis in phloem tissue. Clément et al. (2018) detected relatively high levels of fructose in both the laminae and midveins of *Brassica napus* leaves with higher concentrations in the laminae. Fructose is a plausible source of reducing power for phloem tissue despite the ATP requirements for its catabolism (Fig. 2) and provides the largest fractional contribution to the reducing power in petiolar sieve elements in our model. Additionally, several hexose symporter proteins are expressed equally in both the mesophyll and companion cell transcriptomes (Kim et al. 2021); however, the high fructose uptake, as well as the dark phase glucose uptake, by companion cells is unexpected. Removing the hexose import reactions from our companion cell model resulted in an increase in sucrose import corresponding to the fructose import flux in both the light and dark phases and a decrease in overall phloem transport rate indicating that it is more efficient to import hexoses into the phloem than to break down imported sucrose (Supplemental Table S3).

Our assumption that sieve element maintenance and protein turnover costs would be similar to those of mesophyll cells on a per cell basis is likely to be an overestimate, especially given their relative lack of organelles and protein-synthesizing machinery. Despite this, our model analysis indicates that the phloem transport rate of the system is maximized when the sieve elements are able to catabolize sugars present in phloem sap and fulfil the energy costs associated with keeping themselves alive and intact. Given the role of mitochondria in this energetic metabolism, it is relevant to consider that sieve element mitochondria are more rudimentary than those found in other cell types (Behnke et al. 1990; Liu et al. 2022). However, the available evidence suggests that they are still present and metabolically active in mature sieve elements (McGivern 1957; Esau and Cronshaw 1968; Lee et al. 1971; Moninger et al. 1993) and this indicates that they do perform some function within these cells. Whether they are capable of providing the energy needs of sieve elements and how much energy sieve elements actually require to meet their maintenance costs is not known.

To test the effect of sieve element maintenance costs, we re-ran the model with the cost halved. This reduces the load on the sieve element mitochondria, reducing both the sucrose synthase and invertase fluxes within them, and almost doubles the total phloem transport rate of the model (Supplemental Table S4). None of the central conclusions we have made are affected, but there are inevitably quantitative differences in the model as a result of reduced sieve element maintenance cost. For example, there is a much lower reliance on hexose uptake by companion cells in both the light and dark phases, a lower companion cell PMF requirement (due to the reduced need for hexose import) so less flux through H^+^-PP_i_ase (although this remains the dominant companion cell proton pump), and less energetic pressure on companion cell chloroplasts allowing them the flexibility to fix carbon (although the majority of photosynthetic energy is still used elsewhere with each cell only fixing 2% of the carbon a mesophyll cell is predicted to fix). This indicates a potentially substantial drain on plant resources depending on the metabolic activity in sieve elements which at a minimum must be enough to keep them alive and sustain active retrieval of leaked photoassimilates.

Proteomic surveys of phloem sap have found measurable amounts of proteins belonging to both amino acid synthesis and carbohydrate catabolism (Batailler et al. 2012; Carella et al. 2016; Liu et al. 2022). However, a recent proteome analysis of sieve elements in *N. tabacum* showed a lack of key protein subunits necessary for several amino acid synthesis reactions (Supplemental Table S5). Removal of these reactions from sieve elements in our model shifts arginine, lysine, and valine biosynthesis from sieve elements to being solely synthesized within mesophyll cells. Additionally, ∼10% of model reactions could not be matched to protein fragments in the Liu et al. (2022) data set. Removal of these from modeled sieve elements further reduces the total phloem transport rate of the model. Otherwise, the model remained largely unchanged (Supplemental Table S6).

### Comparison between model solution and Kim et al. interpretation

While not all metabolic pathways identified in the transcriptome data set were present in our model, there was general agreement between our model predictions and those made solely from the transcriptome data (Kim et al. 2021) with the exception of amino acid synthesis and degradation pathways (Supplemental Table S7). Kim et al. (2021) predicted greater biosynthesis of glutamine and arginine in phloem cells but lower biosynthesis of leucine, methionine, lysine, valine, glycine, isoleucine, histidine, and phenylalanine in phloem cells compared to the average leaf cell. We see greater biosynthesis of glutamine and arginine in phloem cells in our model on a per cell basis, but our model also predicts higher biosynthesis of lysine and histidine for the reasons we mentioned earlier (Fig. 4). This difference could be because of an overestimation of companion cell and sieve element metabolic capacity in our model but is also a plausible method of transferring reducing power to phloem cells. Of the other pathways predicted by Kim et al. to have higher activity in companion cells, most of them have higher activity in the dark phase but lower activity in the light phase in our model solution. During photosynthesis (in the light phase) the metabolic flux of most active pathways is elevated in mesophyll cells; however, at night, in many of these pathways (nonoxidative pentose phosphate pathway, glycolysis, TCA cycle, aerobic respiration), companion cells are much more active than mesophyll cells. The high transcriptome abundance for enzymes in these pathways could potentially be a result of the time of measurement or an indication of the higher enzyme concentration requirements to ensure adequate reaction flux in the dark phase.

### Flexible distribution of reactions between cells of the phloem

Due to the free transport of metabolites via the symplast between companion cells and sieve elements in our model, solutions in which a reaction occurs in the companion cells are equivalent in “cost” to solutions in which the same reaction occurs in the sieve elements instead. For example, the pyrophosphate-generating loop depicted in Fig. 2 could happen almost entirely within companion cells with no effect on modeled phloem transport rate. However, maintenance costs in the sieve elements require that at least some ATP- and NADPH-generating reactions must occur within them. It is due to this that the model solution suggests that the most efficient cells to produce the histidine, leucine, tyrosine, and valine required for phloem transport rate are sieve elements as they are produced from products of the pentose phosphate pathway and glycolysis. The sources of ATP and reducing power in sieve elements are likely to depend on where these amino acids are synthesized, and evidence of either happening in sieve elements would tell us a lot more about the extent of sieve element metabolism. Whether these patterns of metabolite diffusion between the symplasts of phloem cells are achievable would depend on enzyme kinetic and regulatory considerations in order to establish the relevant metabolite concentration gradients, something that is beyond the scope of this work.

### Future perspectives

While FBA models are capable of producing remarkably accurate flux predictions (Williams et al. 2010; Cheung et al. 2014), they are necessarily simplified approximations of the system we are modeling. More data would allow us to make further refinements to our model. Improvements such as higher resolution of the time-dependent changes to the cell metabolism (Töpfer et al. 2020), ideally with transcript data from different times of the day, or greater thermodynamic constraints based on metabolite concentrations might reveal further aspects of phloem metabolism that we have overlooked. Additional cell type–specific data, especially from proteomic and metabolomic analysis, would allow a more detailed assessment of the distribution of metabolism between the individual cell types.

To test the predictions made here, further investigation into the capabilities of companion cell chloroplasts would be necessary, for example, genetic interventions such as knockout of Rubisco in a companion cell–specific manner. Alternatively, measurements of the metabolite gradients between companion cells and adjacent sieve elements would give us a better idea of the likely directions of symplastic transport and ATP/ADP in companion cell chloroplasts and cytosol under varying light intensities and/or with varying access to sucrose to test the energetic link between them may also point toward the major source of companion cell ATP (De Col et al. 2017).

Overall, we have clarified several unknowns in phloem metabolism, and our work may be the basis for further work to improve the efficiency of phloem loading and hence plant growth rate. This could ultimately be important both in mitigating against rising CO_2_ levels and enhancing crop productivity.

## Materials and methods

Each cell within our model contains a stoichiometric model of core cell metabolism based on the model described in Shameer et al. (2019). The core model is available in SBML at https://github.com/hilaryh/phloem. The metabolic network present within each cell is nearly identical. The only differences are as follows: there is a lack of a vacuole and mRNA turnover reaction in sieve elements; only mesophyll cells and companion cells can exchange sugars and amino acids with the apoplast; companion cells have a H^+^-pyrophosphate pump (AVP1) on their plasma membrane which allows them to break down pyrophosphate to export protons or, conversely, import protons to synthesize pyrophosphate; and sieve elements in the petiole have an additional phloem transport rate reaction that fixes the ratios of amino acids and sucrose.

### Comparative populations of each cell type

The ratio of mesophyll cells to vascular cells in mature Arabidopsis (*A. thaliana*) leaves is ∼1:1 (Pyke et al. 1991). Leaf vascular tissue is composed of xylem, phloem, cambium, and the bundle sheath. The phloem accounts for ∼30% of the vascular tissue in Arabidopsis stems (Collins et al. 2015). Assuming this ratio within vascular tissue remains the same in leaves and that the phloem is composed of sieve elements and companion cells in the ratio 5:1, the ratio of mesophyll cells to sieve elements to companion cells is 20:5:1 in leaves.

Sieve elements are ∼100 *μ*m long, and there are ∼100 in the cross section of a petiole. In a petiole of length 15 mm, we would therefore expect ∼15,000 sieve elements. There are ∼50,000 mesophyll cells in an Arabidopsis leaf (Pyke et al. 1991) so the ratio of mesophyll cells to companion cells to sieve elements to petiolar sieve elements is ∼20:1:5:6. To prevent modeled phloem cells undertaking an implausibly high proportion of leaf tissue metabolism, the absolute values of the upper and lower bounds of each reaction in companion cells and sieve elements were therefore constrained to be less than or equal to the flux through the most active mesophyll cell reaction (usually Photon_ep). Further, the bounds on maintenance fluxes, including protein and mRNA turnover, and carbon uptake constraints were additionally scaled by cell ratios. For example, any reactions with an upper bound of 20 *μ*mol m^−2^ s^−1^ in mesophyll cells had an upper bound of only 1, 5, and 6 *μ*mol m^−2^ s^−1^ in companion cells, sieve elements, and petiole sieve elements, respectively.

### Companion cell chloroplasts

While the influence of chloroplast size on photosynthetic efficiency appears to be complicated (Li et al. 2013; Xiong et al. 2017), companion cells have fewer and smaller chloroplasts than mesophyll cells and there are ∼5% as many companion cells as mesophyll cells in the leaf. We have estimated that companion cell chloroplasts can capture no more than 1% of the photons captured by mesophyll chloroplasts and constrained the companion cell photon uptake reaction (Photon_ep_CC_l) accordingly.

### Protein/RNA turnover

Protein degradation rates in mature Arabidopsis leaves are highly variable, but the degradation rates, *K_d_*, of known metabolic proteins tend to magnitudes of ∼0.1 per day (Li et al. 2017) or ∼10^−6^ per s. The fold change in protein, Δ*P*, over time can be calculated as


$$\Delta P(t)\;=\;e^{-Kdt}\;$$

Δ*P* is then 10^−6^ per s. There is ∼15 mg protein per gram fresh weight (gFW) in Arabidopsis leaves (Piques et al. 2009). Assuming the average molecular weight of each amino acid is 118.9 g mol^−1^ (Hachiya et al. 2007), we arrive at an amino acid density of 0.13 mmol amino acid per gram fresh weight (AA/gFW). The fold change in amino acids is then 1.3 × 10^−7^ mmol AA/gFW/s or 1.1 *µ*mol AA/gFW/day. The maximum estimated rate of protein synthesis is 26 *µ*mol AA/gFW/day, within an order of magnitude of our estimate.

With a fresh weight (FW) to dry weight (DW) ratio of ∼1:50, we estimate the DW protein density to be 6.3 mmol AA/gDW. The fold change in amino acids is then 6.3 × 10^−3^*µ*mol AA/gDW/s. Leaf mass per area is ∼1.5 mg DW cm^−2^ = 15 g DW m^−2^ (Liu et al. 2020). Then, the flux through our protein turnover reaction is 9.45 × 10^−2^*µ*mol AA m^−2^ s^−1^.

The total RNA present in Arabidopsis cells varies throughout the day. On average, it is ∼600 *µ*g/gFW (Piques et al. 2009). The half-life of mRNA is ∼107 min (Sorenson et al. 2018; Szabo et al. 2020). Thus, the fold change in mRNA is 10^−4^ s^−1^. The average molecular weight of a ribonucleotide is 500 Da. Then, the flux through our RNA turnover reactions should be 0.9 *µ*mol m^−2^ s^−1^.

### Diel modeling

FBA models are typically used to model steady-state fluxes. As there are clearly at least 2 major metabolic modes in leaf photosynthesis and phloem loading, the model was doubled to predict a steady-state metabolism in both the light (day) and dark (night) phases of the plant's growth that approximates the average metabolism in leaf cell types over the day and night. These 2 temporal phases are treated as separated compartments within the model and connected by accumulation reactions that mimic the build-up of starch, sucrose, nitrate, malate, and amino acids during either the light or dark phase for use in the opposite temporal phase. The metabolism in each of the 2 phases was handled simultaneously as part of a single optimization problem using the parsimonious solver in CobraPy (Ebrahim et al. 2013). This solver returns a vector of reaction fluxes that conserves mass and charge in the system; maximizes our objective, phloem transport flux; and has a minimal sum of fluxes.

### Weighting FBA model with transcriptome data

We used cell-specific transcriptome data published by Kim et al. (2021) to weight model solutions toward those more likely to represent in vivo cell metabolism using a modification of the RegrEx method developed by Robaina Estévez and Nikoloski (2014) and Robaina-Estévez et al. (2017). For companion cell and sieve element reactions where companion cells synthesize the transcripts for both cell types, the objective was to minimize the sum of the difference between the reaction fluxes in each cell type and their corresponding transcript abundance in companion cells. In mesophyll cells, we wanted to minimize the sum of the differences between each reaction in just that cell type and its corresponding transcript abundance. This objective can be written as:


$$\begin{array}{rl} H\;= & \mathrm{minimise}\\ & \left(\sum_{i\in D_{rxns}}\|r_{MC}d_{i_{MC}}-v_{i_{MC}}\|+\|r_{CC}d_{i_{CC}}\;-\;(v_{i_{CC}}\;+v_{i_{SE}}+v_{i_{\;pSE}})\|\right) \end{array}$$

where *r_cell_* is the ratio of the cell type in leaves (i.e. *r_MC_* = 20, *r_CC_* = 1), *d_icell_* is the normalized transcript abundance of reaction *i* in *cell* and *v_icell_* is the flux through reaction *i* in *cell*, and *D_rxns_* is the set of reactions for which we have transcript abundance data. We prioritized the similarity to transcriptome data and then identified a solution with the minimum sum of fluxes (parsimonious) within that solution space so that our objective, *G*, was


$$G=\mathrm{minimise}\left(\underset{i\in rxns}{\sum }\;\|v_{i}\|\right)\;\mathrm{given}\;H\;\mathrm{minimised}$$

where *rxns* includes all reactions in the model. We enforce a minimum phloem transport rate and cell maintenance cost so important pathways in these outputs may have a higher minimum flux than the transcript abundance would otherwise necessitate.

To check the stability of our observations, flux variability analysis (FVA) (Mahadevan and Schilling 2003) was performed on both the transcriptome-weighted and parsimonious versions of our model. The FBA solutions were generated using gurobipy and the COBRAPy package (Ebrahim et al. 2013). Python code required to reproduce the results in this article can be found at https://github.com/hilaryh/phloem.

## Supplementary Material

kiad154_Supplementary_Data

## Supplemental data

The following materials are available in the online version of this article.

**Supplemental Figure S1.** The principal reactions involved in ATP and PPi biosynthesis and the movement of energy and reducing power from chloroplast to the cytosol and mitochondrion in the parsimonious solution.

**Supplemental Figure S2.** ATP budgets for each cell in the RegrEx model solution.

**Supplemental Figure S3.** PPi budgets for each cell in the RegrEx model solution.

**Supplemental Figure S4.** NADH budgets for each cell in the RegrEx model solution.

**Supplemental Table S1.** Model reactions, their stoichiometries, and fluxes in the parsimonious and RegrEx model solutions as well as flux variability analysis ranges.

**Supplemental Table S2.** Predicted reaction fluxes when AVP1 activity is restricted to only 10% of the optimal.

**Supplemental Table S3.** Predicted reaction fluxes when hexose uptake is prevented in model companion cells.

**Supplemental Table S4.** Predicted reaction fluxes when sieve element maintenance costs are halved.

**Supplemental Table S5.** Mapping of *N. tabacum* sieve element proteome data onto model reactions.

**Supplemental Table S6.** Predicted reaction fluxes when proteins not found in *N. tabacum* data set are removed from modeled sieve elements.

**Supplemental Table S7.** Comparison of pathway activity between transcriptome data–based and transcriptome-weighted model predictions.

## References

[kiad154-B1] Ambasht PK , Kayastha AM. Plant pyruvate kinase. Biol Plant. 2002:45(1):1–10. 10.1023/A:1015173724712

[kiad154-B2] Ayre BG , Keller F, Turgeon R. Symplastic continuity between companion cells and the translocation stream: long-distance transport is controlled by retention and retrieval mechanisms in the phloem. Plant Physiol. 2003:131(4):1518–1528. 10.1104/pp.012054 12692312 PMC166911

[kiad154-B3] Bailleul B , Berne N, Murik O, Petroutsos D, Prihoda J, Tanaka A, Villanova V, Bligny R, Flori S, Falconet D, et al Energetic coupling between plastids and mitochondria drives CO_2_ assimilation in diatoms. Nature. 2015:524(7565):366–369. 10.1038/nature14599 26168400

[kiad154-B4] Bao A-K , Wang S-M, Wu G-Q, Xi J-J, Zhang J-L, Wang C-M. Overexpression of the Arabidopsis H^+^-PPase enhanced resistance to salt and drought stress in transgenic alfalfa (*Medicago sativa* L.). Plant Sci. 2009:176(2):232–240. 10.1016/j.plantsci.2008.10.009

[kiad154-B5] Barratt DHP , Derbyshire P, Findlay K, Pike M, Wellner N, Lunn J, Feil R, Simpson C, Maule AJ, Smith AM. Normal growth of Arabidopsis requires cytosolic invertase but not sucrose synthase. Proc Natl Acad Sci U S A. 2009:106(31):13124–13129. 10.1073/pnas.0900689106 19470642 PMC2722301

[kiad154-B6] Barratt DHP , Kölling K, Graf A, Pike M, Calder G, Findlay K, Zeeman SC, Smith AM. Callose synthase GSL7 is necessary for normal phloem transport and inflorescence growth in Arabidopsis. Plant Physiol. 2011:155(1):328–341. 10.1104/pp.110.166330 21098675 PMC3075753

[kiad154-B7] Batailler B , Lemaître T, Vilaine F, Sanchez C, Renard D, Cayla T, Beneteau J, Dinant S. Soluble and filamentous proteins in Arabidopsis sieve elements. Plant Cell Environ. 2012:35(7):1258–1273. 10.1111/j.1365-3040.2012.02487.x 22292537

[kiad154-B8] Behnke D . Distribution and evolution of forms and types of sieve-element plastids in the dicotyledons. Aliso. 1991:13(1):167–182. 10.5642/aliso.19911301.06

[kiad154-B9] Behnke HD , Sjolund RD; International Botanical Congress. Sieve elements: comparative structure, induction and development. Berlin; London: Springer-Verlag; 1990.

[kiad154-B10] Bollard EG . Transport in the xylem. Annu Rev Plant Physiol. 1960:11(1):141–166. 10.1146/annurev.pp.11.060160.001041

[kiad154-B11] Braun DM , Wang L, Ruan Y-L. Understanding and manipulating sucrose phloem loading, unloading, metabolism, and signalling to enhance crop yield and food security. J Exp Bot. 2014:65(7):1713–1735. 10.1093/jxb/ert416 24347463

[kiad154-B12] Carella P , Merl-Pham J, Wilson DC, Dey S, Hauck SM, Corina Vlot A, Cameron RK. Comparative proteomics analysis of phloem exudates collected during the induction of systemic acquired resistance. Plant Physiol. 2016:171(2):1495–1510. 10.1104/pp.16.00269 27208255 PMC4902610

[kiad154-B13] Cayla T , Batailler B, Le Hir R, Revers F, Anstead JA, Thompson GA, Grandjean O, Dinant S. Live imaging of companion cells and sieve elements in *Arabidopsis* leaves. PLoS One. 2015:10(2):e0118122. 10.1371/journal.pone.0118122 PMC4340910 25714357

[kiad154-B14] Cheung CYM , George Ratcliffe R, Sweetlove LJ. A method of accounting for enzyme costs in flux balance analysis reveals alternative pathways and metabolite stores in an illuminated Arabidopsis leaf. Plant Physiol. 2015:169(3):1671–1682. 10.1104/pp.15.00880 26265776 PMC4634065

[kiad154-B15] Cheung CYM , Poolman MG, David AF, George Ratcliffe R, Sweetlove LJ. A diel flux balance model captures interactions between light and dark metabolism during day–night cycles in C_3_ and Crassulacean acid metabolism leaves. Plant Physiol. 2014:165(2):917–929. 10.1104/pp.113.234468 24596328 PMC4044858

[kiad154-B16] Cheung CYM , Williams TCR, Poolman MG, Fell DA, George Ratcliffe R, Sweetlove LJ. A method for accounting for maintenance costs in flux balance analysis improves the prediction of plant cell metabolic phenotypes under stress conditions. Plant J. 2013:75(6):1050–1061. 10.1111/tpj.12252 23738527

[kiad154-B17] Clément G , Moison M, Soulay F, Reisdorf-Cren M, Masclaux-Daubresse C. Metabolomics of laminae and midvein during leaf senescence and source–sink metabolite management in *Brassica napus* L. leaves. J Exp Bot. 2018:69(4):891–903. 10.1093/jxb/erx253 28992054 PMC5853214

[kiad154-B18] Collins C , Maruthi NM, Jahn CE. CYCD3 D-type cyclins regulate cambial cell proliferation and secondary growth in Arabidopsis. J Exp Bot. 2015:66(15):4595–4606. 10.1093/jxb/erv218 26022252 PMC4507761

[kiad154-B19] Davies JM . The bioenergetics of vacuolar H^+^ pumps. In: Leigh RA, Sanders D, Callow JA, editors. The plant vacuole: Advances in botanical research. Vol. 25. London: Academic Press; 1997. p. 339–363.

[kiad154-B20] Davies JM , Poole RJ, Sanders D. The computed free energy change of hydrolysis of inorganic pyrophosphate and ATP: apparent significance. for inorganic-pyrophosphate-driven reactions of intermediary metabolism. Biochim Biophys Acta. 1993:1141(1):29–36. 10.1016/0005-2728(93)90185-I

[kiad154-B21] De Col V , Fuchs P, Nietzel T, Elsässer M, Voon CP, Candeo A, Seeliger I, Fricker MD, Grefen C, Møller IM, et al ATP sensing in living plant cells reveals tissue gradients and stress dynamics of energy physiology. eLife. 2017:6:e26770. 10.7554/eLife.26770 PMC5515573 28716182

[kiad154-B22] Dekker JP , Boekema EJ. Supramolecular organization of thylakoid membrane proteins in green plants. Biochim Biophys Acta. 2005:1706(1–2):12–39. 10.1016/j.bbabio.2004.09.009 15620363

[kiad154-B23] Dekker JP , Germano M, van Roon H, Boekema EJ. Photosystem II solubilizes as a monomer by mild detergent treatment of unstacked thylakoid membranes. Photosynth Res. 2002:72(2):203–210. 10.1023/A:1016188818591 16228518

[kiad154-B24] De Schepper V , De Swaef T, Bauweraerts I, Steppe K. Phloem transport: a review of mechanisms and controls. J Exp Bot. 2013:64(16):4839–4850. 10.1093/jxb/ert302 24106290

[kiad154-B25] DeWitt ND , Sussman MR. Immunocytological localization of an epitope-tagged plasma membrane proton pump (H(+)-ATPase) in phloem companion cells. Plant Cell. 1995:7(12):2053–2067. 10.1105/tpc.7.12.2053 8718619 PMC161061

[kiad154-B26] Donahue RA , Poulson ME, Edwards GE. A method for measuring whole plant photosynthesis in *Arabidopsis thaliana*. Photosyn Res. 1997:52(3):263–269. 10.1023/A:1005834327441

[kiad154-B27] Ebrahim A , Lerman JA, Palsson BO, Hyduke DR. COBRApy: COnstraints-Based Reconstruction and Analysis for Python. BMC Syst Biol. 2013:7(1):74. 10.1186/1752-0509-7-74 23927696 PMC3751080

[kiad154-B28] Esau K . Development and structure of the phloem tissue. Bot Rev. 1939:5(7):373–432. 10.1007/BF02878295

[kiad154-B29] Esau K , Cheadle VI, Gifford EM. Comparative structure and possible trends of specialization of the phloem. Am J Bot. 1953:40(1):9–19. 10.2307/2438486

[kiad154-B30] Esau K , Cronshaw J. Plastids and mitochondria in the phloem of *Cucurbita*. Can J Bot. 1968:46(7):877–880. 10.1139/b68-116

[kiad154-B31] Fünfgeld MMFF , Wang W, Ishihara H, Arrivault S, Feil R, Smith AM, Stitt M, Lunn JE, Niittylä T. Sucrose synthases are not involved in starch synthesis in Arabidopsis leaves. Nat Plants. 2022:8(5):574–582. 10.1038/s41477-022-01140-y 35484201 PMC9122829

[kiad154-B32] Gaxiola RA , Regmi K, Paez-Valencia J, Pizzio G, Zhang S. Plant H^+^-PPases: reversible enzymes with contrasting functions dependent on membrane environment. Mol Plant. 2016:9(3):317–319. 10.1016/j.molp.2015.09.008 26407528

[kiad154-B33] Gaxiola RA , Sanchez CA, Paez-Valencia J, Ayre BG, Elser JJ. Genetic manipulation of a “vacuolar” H^+^-PPase: from salt tolerance to yield enhancement under phosphorus-deficient soils. Plant Physiol. 2012:159(1):3–11. 10.1104/pp.112.195701 22434041 PMC3375966

[kiad154-B34] Gonzalez N , De Bodt S, Sulpice R, Jikumaru Y, Chae E, Dhondt S, Van Daele T, De Milde L, Weigel D, Kamiya Y, et al Increased leaf size: different means to an end. Plant Physiol. 2010:153(3):1261–1279. 10.1104/pp.110.156018 20460583 PMC2899902

[kiad154-B35] Goodwin PB . Molecular size limit for movement in the symplast of the Elodea leaf. Planta. 1983:157(2):124–130. 10.1007/BF00393645 24264065

[kiad154-B36] Gould N , Thorpe MR, Pritchard J, Christeller JT, Williams LE, Roeb G, Schurr U, Minchin PEH. AtSUC2 has a role for sucrose retrieval along the phloem pathway: evidence from carbon-11 tracer studies. Plant Sci. 2012:188–189:97–101. 10.1016/j.plantsci.2011.12.018 22525249

[kiad154-B37] Gutiérrez-Luna FM , Hernández-Domínguez EE, Valencia-Turcotte LG, Rodríguez-Sotres R. Review: “pyrophosphate and pyrophosphatases in plants, their involvement in stress responses and their possible relationship to secondary metabolism”. Plant Sci. 2018:267:11–19. 10.1016/j.plantsci.2017.10.016 29362089

[kiad154-B38] Hachiya T , Terashima I, Noguchi K. Increase in respiratory cost at high growth temperature is attributed to high protein turnover cost in Petunia x hybrida petals. Plant Cell Environ. 2007:30(10):1269–1283. 10.1111/j.1365-3040.2007.01701.x 17727417

[kiad154-B39] Hafke JB , van Amerongen J-K, Kelling F, Furch ACU, Gaupels F, van Bel AJE. Thermodynamic battle for photosynthate acquisition between sieve tubes and adjoining parenchyma in transport phloem. Plant Physiol. 2005:138(3):1527–1537. 10.1104/pp.104.058511 15980202 PMC1176423

[kiad154-B40] Haritatos E , Medville R, Turgeon R. Minor vein structure and sugar transport in *Arabidopsis thaliana*. Planta. 2000:211(1):105–111. 10.1007/s004250000268 10923710

[kiad154-B41] Haruta M , Burch HL, Nelson RB, Barrett-Wilt G, Kline KG, Mohsin SB, Young JC, Otegui MS, Sussman MR. Molecular characterization of mutant Arabidopsis plants with reduced plasma membrane proton pump activity. J Biol Chem. 2010:285(23):17918–17929. 10.1074/jbc.M110.101733 20348108 PMC2878554

[kiad154-B42] Igamberdiev AU , Kleczkowski LA. Pyrophosphate as an alternative energy currency in plants. Biochem J. 2021:478(8):1515–1524. 10.1042/BCJ20200940 33881486

[kiad154-B43] Khadilkar AS , Yadav UP, Salazar C, Shulaev V, Paez-Valencia J, Pizzio GA, Gaxiola RA, Ayre BG. Constitutive and companion cell-specific overexpression of *AVP1*, encoding a proton-pumping pyrophosphatase, enhances biomass accumulation, phloem loading, and long-distance transport. Plant Physiol. 2016:170(1):401–414. 10.1104/pp.15.01409 26530315 PMC4704589

[kiad154-B44] Kim J-Y , Symeonidi E, Pang TY, Denyer T, Weidauer D, Bezrutczyk M, Miras M, Zöllner N, Hartwig T, Wudick MM, et al Distinct identities of leaf phloem cells revealed by single cell transcriptomics. Plant Cell. 2021:33(3):511–530. 10.1093/plcell/koaa060 33955487 PMC8136902

[kiad154-B45] Knoblauch M , Knoblauch J, Mullendore DL, Savage JA, Babst BA, Beecher SD, Dodgen AC, Jensen KH, Michele Holbrook N. Testing the Münch hypothesis of long distance phloem transport in plants. eLife. 2016:5:e15341. 10.7554/eLife.15341 PMC4946904 27253062

[kiad154-B46] Lan P , Li W, Schmidt W. Complementary proteome and transcriptome profiling in phosphate-deficient Arabidopsis roots reveals multiple levels of gene regulation. Mol Cell Proteomics. 2012:11(11):1156. 10.1074/mcp.M112.020461 22843991 PMC3494196

[kiad154-B47] Langhans M , Ratajczak R, Lützelschwab M, Michalke W, Wächter R, Fischer-Schliebs E, Ullrich CI. Immunolocalization of plasma-membrane H^+^-ATPase and tonoplast-type pyrophosphatase in the plasma membrane of the sieve element–companion cell complex in the stem of *Ricinus communis* L. Planta. 2001:213(1):11–19. 10.1007/s004250000475 11523647

[kiad154-B48] Lee DR , Arnold DC, Fensom DS. Some microscopical observations of functioning sieve tubes of Heracleum using Nomarski optics. J Exp Bot. 1971:22(1):25–38. 10.1093/jxb/22.1.25

[kiad154-B49] Li L , Nelson CJ, Trösch J, Castleden I, Huang S, Harvey Millar A. Protein degradation rate in *Arabidopsis thaliana* leaf growth and development. Plant Cell. 2017:29(2):207–228. 10.1105/tpc.16.00768 28138016 PMC5354193

[kiad154-B50] Li Y , Ren B, Ding L, Shen Q, Peng S, Guo S. Does chloroplast size influence photosynthetic nitrogen use efficiency? PLoS One. 2013:8(4):e62036. 10.1371/journal.pone.0062036 PMC3631174 23620801

[kiad154-B51] Li Z , Wakao S, Fischer BB, Niyogi KK. Sensing and responding to excess light. Annu Rev Plant Biol. 2009:60(1):239–260. 10.1146/annurev.arplant.58.032806.103844 19575582

[kiad154-B52] Liu P-C , Peacock WJ, Wang L, Furbank R, Larkum A, Dennis ES, Raines C. Leaf growth in early development is key to biomass heterosis in Arabidopsis. J Exp Bot. 2020:71(8):2439–2450. 10.1093/jxb/eraa006 31960925 PMC7178430

[kiad154-B53] Liu Y , Vasina VV, Kraner ME, Peters WS, Sonnewald U, Knoblauch M. Proteomics of isolated sieve tubes from *Nicotiana tabacum*: sieve element–specific proteins reveal differentiation of the endomembrane system. Proc Natl Acad Sci U S A. 2022:119(1):e2112755119. 10.1073/pnas.2112755119 PMC8740716 34983847

[kiad154-B54] Mahadevan R , Schilling CH. The effects of alternate optimal solutions in constraint-based genome-scale metabolic models. Metab Eng. 2003:5(4):264–276. 10.1016/j.ymben.2003.09.002 14642354

[kiad154-B55] McGivern M . Mitochondria and plastids in sieve-tube cells. Am J Bot. 1957:44(1):37–48. 10.1002/j.1537-2197.1957.tb08207.x

[kiad154-B56] Moninger T , Wang Q, Sjolund R. Rhodamine 123 labelling of mitochondria in sieve elements I. Localization with confocal microscopy in plant tissue culture phloem. Acta Microsc. 1993:2(1):93–98. https://acta-microscopica.org/acta/article/view/30

[kiad154-B57] Müller P , Li X-P, Niyogi KK. Non-photochemical quenching. A response to excess light energy. Plant Physiol. 2001:125(4):1558–1566. 10.1104/pp.125.4.1558 11299337 PMC1539381

[kiad154-B58] Münch E . Die Stoffbewegungen in der Pflanze. Jena: G. Fischer; 1930.

[kiad154-B59] Nikoloski Z , Perez-Storey R, Sweetlove LJ. Inference and prediction of metabolic network fluxes. Plant Physiol. 2015:169(3):1443–1455. 10.1104/pp.15.01082 26392262 PMC4634083

[kiad154-B60] Otero S , Helariutta Y. Companion cells: a diamond in the rough. J Exp Bot. 2017:68(1):71–78. 10.1093/jxb/erw392 27811001

[kiad154-B61] Paez-Valencia J , Patron-Soberano A, Rodriguez-Leviz A, Sanchez-Lares J, Sanchez-Gomez C, Valencia-Mayoral P, Diaz-Rosas G, Gaxiola R. Plasma membrane localization of the type I H^+^-PPase AVP1 in sieve element–companion cell complexes from *Arabidopsis thaliana*. Plant Sci. 2011:181(1):23–30. 10.1016/j.plantsci.2011.03.008 21600394

[kiad154-B62] Paramonova NV , Krasavina MS, Sokolova SV. Ultrastructure of chloroplasts in phloem companion cells and mesophyll cells as related to the stimulation of sink activity by cytokinins. Russ J Plant Physiol. 2002:49(2):187–195. 10.1023/A:1014893221505

[kiad154-B63] Pazhamala LT , Kudapa H, Weckwerth W, Harvey Millar A, Varshney RK. Systems biology for crop improvement. Plant Genome. 2020:14(2):e20098. 10.1002/tpg2.20098 PMC12806876 33949787

[kiad154-B64] Peng Z , He S, Gong W, Xu F, Pan Z, Jia Y, Geng X, Du X. Integration of proteomic and transcriptomic profiles reveals multiple levels of genetic regulation of salt tolerance in cotton. BMC Plant Biol. 2018:18(1):128. 10.1186/s12870-018-1350-1 29925319 PMC6011603

[kiad154-B65] Piques M , Schulze WX, Höhne M, Usadel B, Gibon Y, Rohwer J, Stitt M. Ribosome and transcript copy numbers, polysome occupancy and enzyme dynamics in Arabidopsis. Mol Syst Biol. 2009:5(1):314. 10.1038/msb.2009.68 19888209 PMC2779082

[kiad154-B66] Pizzio GA , Paez-Valencia J, Khadilkar AS, Regmi K, Patron-Soberano A, Zhang S, Sanchez-Lares J, Furstenau T, Li J, Sanchez-Gomez C, et al Arabidopsis type I proton-pumping pyrophosphatase expresses strongly in phloem, where it is required for pyrophosphate metabolism and photosynthate partitioning. Plant Physiol. 2015:167(4):1541–1553. 10.1104/pp.114.254342 25681328 PMC4378163

[kiad154-B67] Primo C , Pizzio GA, Yang J, Gaxiola RA, Scholz-Starke J, Hirschi KD. Plant proton pumping pyrophosphatase: the potential for its pyrophosphate synthesis activity to modulate plant growth. Plant Biol. 2019:21(6):989–996. 10.1111/plb.13007 31081197

[kiad154-B68] Pyke KA , Marrison JL, Leech AM. Temporal and spatial development of the cells of the expanding first leaf of *Arabidopsis thaliana* (L.) Heynh. J Exp Bot. 1991:42(11):1407–1416. 10.1093/jxb/42.11.1407

[kiad154-B69] Robaina-Estévez S , Daloso DM, Zhang Y, Fernie AR, Nikoloski Z. Resolving the central metabolism of Arabidopsis guard cells. Sci Rep. 2017:7(1):8307. 10.1038/s41598-017-07132-9 28814793 PMC5559522

[kiad154-B70] Robaina Estévez S , Nikoloski Z. Generalized framework for context-specific metabolic model extraction methods. Front Plant Sci. 2014:5:491. 10.3389/fpls.2014.00491 25285097 PMC4168813

[kiad154-B71] Robaina Estévez S , Nikoloski Z. Context-specific metabolic model extraction based on regularized least squares optimization. PLoS One. 2015:10(7):e0131875. 10.1371/journal.pone.0131875 PMC4497637 26158726

[kiad154-B72] Robertson WR , Clark K, Young JC, Sussman MR. An *Arabidopsis thaliana* plasma membrane proton pump is essential for pollen development. Genetics. 2004:168(3):1677. 10.1534/genetics.104.032326 15579716 PMC1448765

[kiad154-B73] Rocha Façanha A , de Meis L. Reversibility of H^+^-ATPase and H^+^-pyrophosphatase in tonoplast vesicles from maize coleoptiles and seeds. Plant Physiol. 1998:116(4):1487–1495. 10.1104/pp.116.4.1487 9536067 PMC35057

[kiad154-B74] Scheunemann M , Brady SM, Nikoloski Z. Integration of large-scale data for extraction of integrated Arabidopsis root cell-type specific models. Sci Rep. 2018:8(1):7919. 10.1038/s41598-018-26232-8 29784955 PMC5962614

[kiad154-B75] Schilling RK , Tester M, Marschner P, Plett DC, Roy SJ. AVP1: one protein, many roles. Trends Plant Sci. 2017:22(2):154–162. 10.1016/j.tplants.2016.11.012 27989652

[kiad154-B76] Scholz-Starke J , Primo C, Yang J, Kandel R, Gaxiola RA, Hirschi KD. The flip side of the Arabidopsis type I proton-pumping pyrophosphatase (AVP1): using a transmembrane H^+^ gradient to synthesize pyrophosphate. J Biol Chem. 2019:294(4):1290–1299. 10.1074/jbc.RA118.006315 30510138 PMC6349097

[kiad154-B77] Schwender J , König C, Klapperstück M, Heinzel N, Munz E, Hebbelmann I, Hay JO, Denolf P, De Bodt S, Redestig H, et al Transcript abundance on its own cannot be used to infer fluxes in central metabolism. Front Plant Sci. 2014:5:668. 10.3389/fpls.2014.00668 25506350 PMC4246676

[kiad154-B78] Segami S , Asaoka M, Kinoshita S, Fukuda M, Nakanishi Y, Maeshima M. Biochemical, structural and physiological characteristics of vacuolar H^+^-pyrophosphatase. Plant Cell Physiol. 2018:59(7):1300–1308. 10.1093/pcp/pcy054 29534212

[kiad154-B79] Seufferheld M , Lea CR, Vieira M, Oldfield E, Docampo R. The H^+^-pyrophosphatase of *Rhodospirillum rubrum* is predominantly located in polyphosphate-rich acidocalcisomes. J Biol Chem. 2004:279(49):51193–51202. 10.1074/jbc.M406099200 15371423

[kiad154-B80] Shameer S , Baghalian K, Maurice Cheung CY, George Ratcliffe R, Sweetlove LJ. Computational analysis of the productivity potential of CAM. Nat Plants. 2018:4(3):165–171. 10.1038/s41477-018-0112-2 29483685

[kiad154-B81] Shameer S , George Ratcliffe R, Sweetlove LJ. Leaf energy balance requires mitochondrial respiration and export of chloroplast NADPH in the light. Plant Physiol. 2019:180(4):1947–1961. 10.1104/pp.19.00624 31213510 PMC6670072

[kiad154-B82] Sonnewald U . Expression of *E. coli* inorganic pyrophosphatase in transgenic plants alters photoassimilate partitioning. Plant J. 1992:2(4):571–581. 10.1046/j.1365-313X.1992.t01-26-00999.x 1344891

[kiad154-B83] Sorenson RS , Deshotel MJ, Johnson K, Adler FR, Sieburth LE. Arabidopsis MRNA decay landscape arises from specialized RNA decay substrates, decapping-mediated feedback, and redundancy. Proc Natl Acad Sci U S A. 2018:115(7):E1485–E1494. 10.1073/pnas.1712312115 29386391 PMC5816150

[kiad154-B84] Stadler R , Wright KM, Lauterbach C, Amon G, Gahrtz M, Feuerstein A, Oparka KJ, Sauer N. Expression of GFP-fusions in Arabidopsis companion cells reveals non-specific protein trafficking into sieve elements and identifies a novel post-phloem domain in roots. Plant J. 2005:41(2):319–331. 10.1111/j.1365-313X.2004.02298.x 15634207

[kiad154-B85] Strasburger E . Ueber den bau und die verrichtungen der leitungsbahnen in den pflanzen. Germany: G. Fischer; 1891.

[kiad154-B86] Sweetlove L , George Ratcliffe R. Flux-balance modeling of plant metabolism. Front Plant Sci. 2011:2:38. 10.3389/fpls.2011.00038 22645533 PMC3355794

[kiad154-B87] Szabo EX , Reichert P, Lehniger M-K, Ohmer M, de Francisco Amorim M, Gowik U, Schmitz-Linneweber C, Laubinger S. Metabolic labeling of RNAs uncovers hidden features and dynamics of the Arabidopsis transcriptome. Plant Cell. 2020:32(4):871–887. 10.1105/tpc.19.00214 32060173 PMC7145469

[kiad154-B88] Taiz L . Plant physiology. 5th ed. Sunderland (MA): Sinauer Associates; 2010. p. 271–304.

[kiad154-B89] Töpfer N , Braam T, Shameer S, George Ratcliffe R, Sweetlove LJ. Alternative crassulacean acid metabolism modes provide environment-specific water-saving benefits in a leaf metabolic model. Plant Cell. 2020:32(12):3689–3705. 10.1105/tpc.20.00132 33093147 PMC7721317

[kiad154-B90] Van Bel AJE . 9—The low profile directors of carbon and nitrogen economy in plants: parenchyma cells associated with translocation channels. In: Gartner BL, editor. Plant stems, physiological ecology. San Diego: Academic Press; 1995. p. 205–II.

[kiad154-B91] van Bel AJE . Interaction between sieve element and companion cell and the consequences for photoassimilate distribution. Two structural hardware frames with associated physiological software packages in dicotyledons? J Exp Bot. 1996:47(Special Issue):1129–1140. 10.1093/jxb/47.Special_Issue.1129 21245242

[kiad154-B92] van Bel AJE . The phloem, a miracle of ingenuity. Plant Cell Environ. 2003:26(1):125–149. 10.1046/j.1365-3040.2003.00963.x

[kiad154-B93] van Bel AJE . Sieve-pore plugging mechanisms. In: Baluska F Volkmann D, Barlow PW, editors. Cell–cell channels. New York (NY): Springer; 2006. p. 113–118.

[kiad154-B94] van Bel AJE . Plasmodesmata: a history of conceptual surprises. In: Sahi VP, Baluška F, editors. Concepts in cell biology—history and evolution, plant cell monographs. Cham: Springer International Publishing; 2018. p. 221–270.

[kiad154-B95] van Bel AJE . The plant axis as the command centre for (re)distribution of sucrose and amino acids. J Plant Physiol. 2021:265:153488. 10.1016/j.jplph.2021.153488 34416599

[kiad154-B96] van Bel AJE , Ehlers K, Knoblauch M. Sieve elements caught in the act. Trends Plant Sci. 2002:7(3):126–132. 10.1016/S1360-1385(01)02225-7 11906836

[kiad154-B97] Vatén A , Dettmer J, Wu S, Stierhof Y-D, Miyashima S, Yadav SR, Roberts CJ, Campilho A, Bulone V, Lichtenberger R, et al Callose biosynthesis regulates symplastic trafficking during root development. Dev Cell. 2011:21(6):1144–1155. 10.1016/j.devcel.2011.10.006 22172675

[kiad154-B98] Wilkinson TL , Douglas AE. Phloem amino acids and the host plant range of the polyphagous Aphid, *Aphis fabae*. Entomol Exp Appl. 2003:106(2):103–113. 10.1046/j.1570-7458.2003.00014.x

[kiad154-B99] Williams TCR , Poolman MG, Howden AJM, Schwarzlander M, Fell DA, George Ratcliffe R, Sweetlove LJ. A genome-scale metabolic model accurately predicts fluxes in central carbon metabolism under stress conditions. Plant Physiol. 2010:154(1):311–323. 10.1104/pp.110.158535 20605915 PMC2938150

[kiad154-B100] Wolf S , Hématy K, Höfte H. Growth control and cell wall signaling in plants. Annu Rev Plant Biol. 2012:63(1):381–407. 10.1146/annurev-arplant-042811-105449 22224451

[kiad154-B101] Xie B , Hong Z. Unplugging the callose plug from sieve pores. Plant Signal Behav. 2011:6(4):491–493. 10.4161/psb.6.4.14653 21386663 PMC3142375

[kiad154-B102] Xiong D , Huang J, Peng S, Li Y. A few enlarged chloroplasts are less efficient in photosynthesis than a large population of small chloroplasts in *Arabidopsis thaliana*. Sci Rep. 2017:7(1):5782. 10.1038/s41598-017-06460-0 28720786 PMC5515944

[kiad154-B103] Yao D , Gonzales-Vigil E, Mansfield SD. Arabidopsis sucrose synthase localization indicates a primary role in sucrose translocation in phloem. J Exp Bot. 2020:71(6):1858–1869. 10.1093/jxb/erz539 31805187 PMC7242074

[kiad154-B104] You Y , Sawikowska A, Lee JE, Benstein RM, Neumann M, Krajewski P, Schmid M. Phloem companion cell-specific transcriptomic and epigenomic analyses identify MRF1, a regulator of flowering. Plant Cell. 2019:31(2):325–345. 10.1105/tpc.17.00714 30670485 PMC6447005

[kiad154-B105] Zhao R , Dielen V, Kinet J-M, Boutry M. Cosuppression of a plasma membrane H^+^-ATPase isoform impairs sucrose translocation, stomatal opening, plant growth, and male fertility. Plant Cell. 2000:12(4):535–546. 10.1105/tpc.12.4.535 10760242 PMC139851

[kiad154-B106] Zhen RG , Kim EJ, Rea PA. The molecular and biochemical basis of pyrophosphate-energized proton translocation at the vacuolar membrane. In: Leigh RA, Sanders D, Callow JA, editors. The plant vacuole: advances in botanical research. Vol. 25. London: Academic Press; 1997. p. 297–337.

[kiad154-B107] Zrenner R , Salanoubat M, Willmitzer L, Sonnewald U. Evidence of the crucial role of sucrose synthase for sink strength using transgenic potato plants (*Solanum tuberosum L.*). Plant J. 1995:7(1):97–107. 10.1046/j.1365-313X.1995.07010097.x 7894514

