# Supplementary material for: Analysis of companion cell and phloem metabolism using a transcriptome-guided model of Arabidopsis metabolism
Source: Plant Physiol. 2023 Mar 11;192(2):1359–77. doi: 10.1093/plphys/kiad154 (PMC10231466; doi:10.1093/plphys/kiad154)
Supplement: kiad154_Supplementary_Data [file kiad154_supplementary_data.zip › SI.pdf]

Supplementary Information Analysis of companion cell and phloem metabolism using a transcriptome-guided model of metabolic fluxes in an Arabidopsis leaf

Hilary Hunt

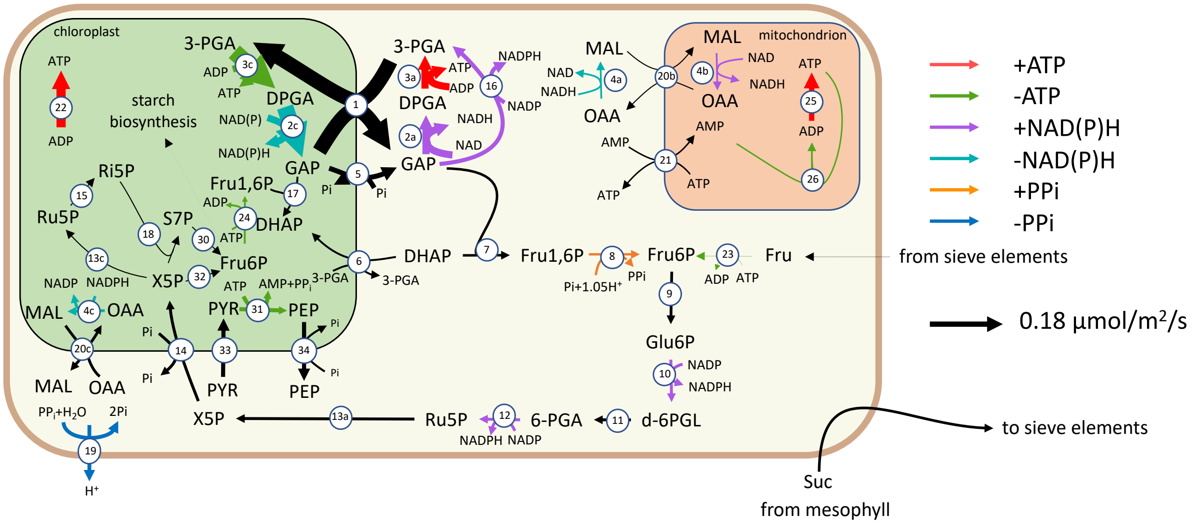

Figure S1: **The principal reactions involved in ATP and PP_i biosynthesis and the movement of energy and reducing power from chloroplast to the cytosol and mitochondrion in the parsimonious solution.** A flux map of the principle reactions involved in companion cells during the light phase. Arrow thickness indicates reaction flux value. Red/green arrows indicate ATP production/consumption, purple/teal arrows indicate NAD(P)H production/consumption, orange/blue arrows indicate PPi production/consumption.

The reactions shown are 1: GAP-3-PGA shuttle, 2: (NADP) GAP dehydrogenase, 3: 3-PGA kinase, 4: Malate dehydrogenase, 5: GAP-Pi shuttle 6: DHAP-3-PGA shuttle, 7: FBP aldolase, 8: FBP phosphotransferase, 9: G6P isomerase, 10: Glu6P dehydrogenase, 11: 6-phosphogluconolactonase, 12: gluconate-6-phosphate dehydrogenase, 13: Ru5P epimerase, 14: X5P-phosphate shuttle, 15: Calvin Cycle, 16: 3-PGA dehydrogenase, 17: triosephosphate isomerase, 18: GDP kinase, 19: H+-pyrophosphatase, 20: Malate-oxaloacetic acid shuttle, 21: ATP-AMP shuttle, 22: GDP kinase, 23: GDP-glucose pyrophosphorylase, 24: Fructose kinase, 25: Mitochondrial ATP synthase, 26: Adenylate kinase

The metabolites shown are GAP: glyceraldehyde-3-phosphate, DPGA: 3-phospho-D-glyceroyl phosphate, 3-PGA: 3-phosphoglycerate, DHAP: Dihydroxy acetone phosphate, MAL: malate, OAA: oxaloacetic acid, Glu1P: glucose-1-phosphate, GDP-Glu: GDP-glucose, Glu6P: glucose-6-phosphate, d-6PGL: 6-phosphoglucono-δ-lactone, 6-PGA: 6-phosphogluconate, Ru5P: ribulose-5-phosphate, X5P: xylulose-5-phosphate, Ri5P: ribose-5-phosphate

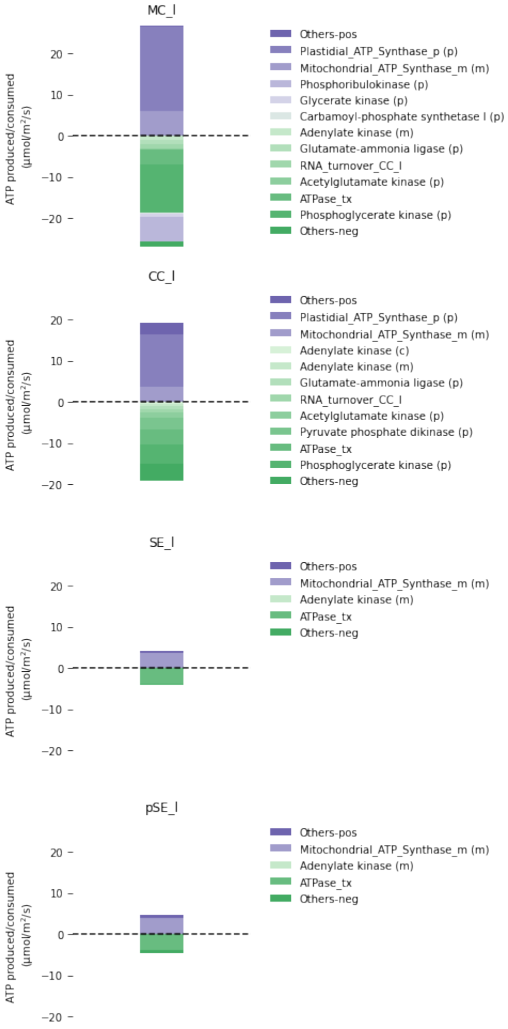

Figure S2: ATP budgets for each cell type in the RegrEx solution. Note: Budgets are scaled according to cell ratios so companion cell fluxes are multiplied by 20, sieve element fluxes are multiplied by 4, and petiole sieve element fluxes are multiplied by 3.3

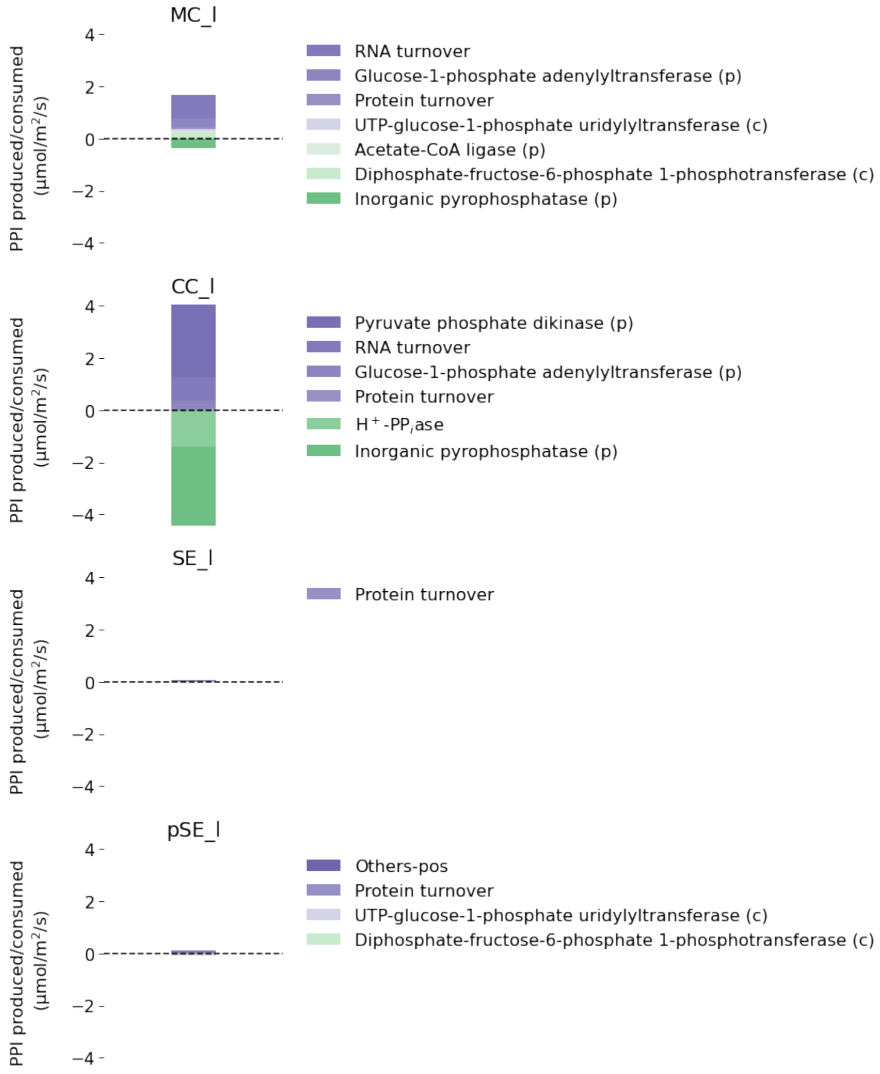

Figure S3: PPI budgets for each cell type in the RegrEx solution. Note: Budgets are scaled according to cell ratios so companion cell fluxes are multiplied by 20, sieve element fluxes are multiplied by 4, and petiole sieve element fluxes are multiplied by 3.3

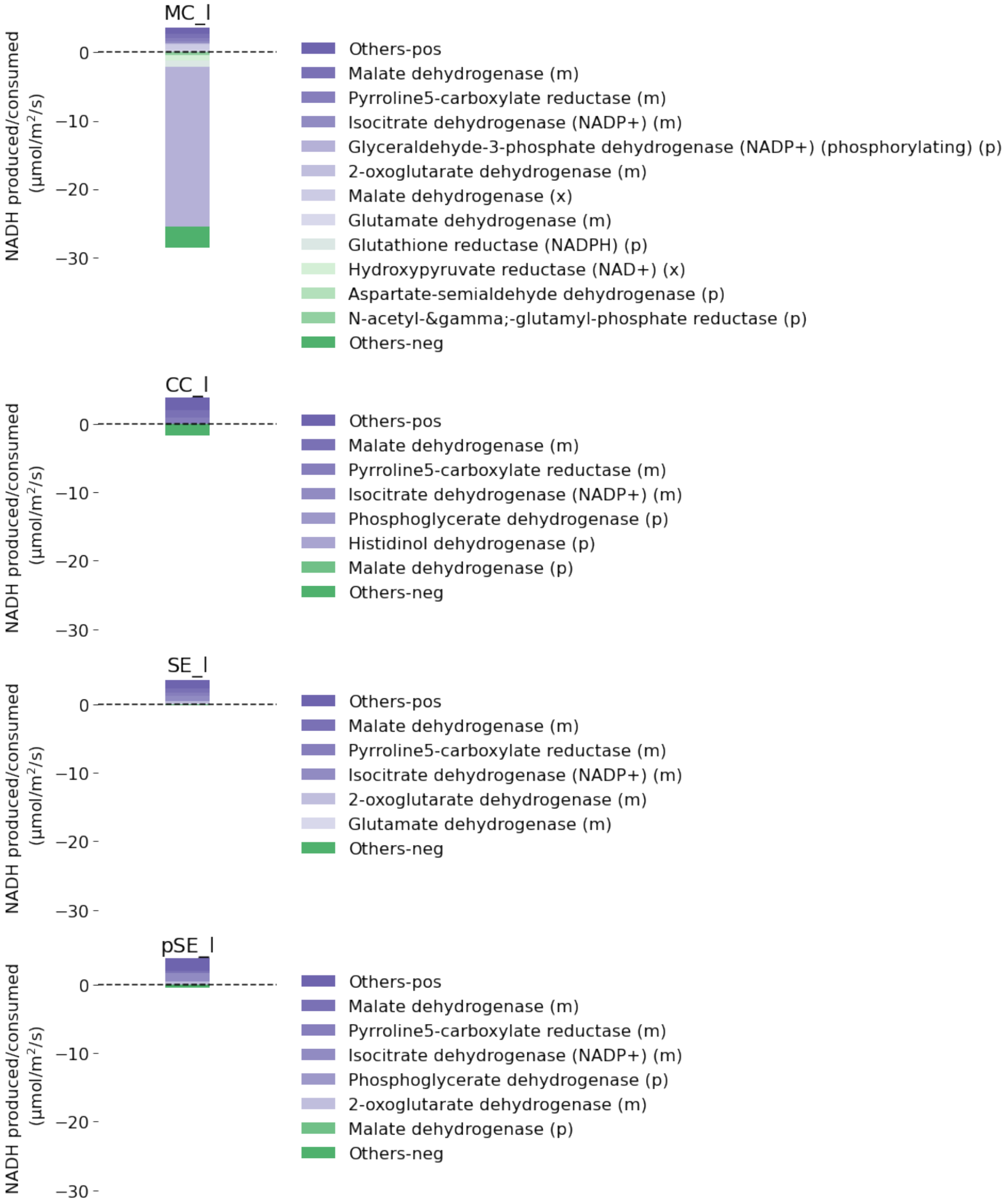

Figure S4: NADH budgets for each cell type in the RegrEx solution. Note: Budgets are scaled according to cell ratios so companion cell fluxes are multiplied by 20, sieve element fluxes are multiplied by 4, and petiole sieve element fluxes are multiplied by 3.3
